# An Anti‐Amyloidogenic Approach to Specifically Block Memory Consolidation in Mice for Therapeutic Intervention

**DOI:** 10.1002/advs.202502541

**Published:** 2026-10-16

**Authors:** Paula López‐García, Kerry R. McGreevy, Maria Eugenia Vaquero, Juan M. Suarez‐Rivero, Antonio Moreno‐Rodríguez, Antonio J. Pérez‐Pulido, Daniel Ramírez de Mingo, Anna Pallé, Helena Akiko Popiel, Andrea Santi, Mario D. Cordero, José Luis Trejo, Yoshitaka Nagai, Mariano Carrión‐Vázquez

**Affiliations:** ^1^ Department of Molecular and Cellular Neurobiology Cajal Neuroscience Center (CNC‐CSIC) (formely Cajal Institute‐CSIC) Madrid Spain; ^2^ Department of Translational Neuroscience Cajal Neuroscience Center (CNC‐CSIC) (formely Cajal Institute‐CSIC) Madrid Spain; ^3^ Department of Molecular Biology and Biochemical Engineering Universidad Pablo de Olavide Seville Spain; ^4^ Andalusian Centre for Developmental Biology (CABD, UPO‐CSIC‐JA) Seville Spain; ^5^ Salk Institute for Biological Studies La Jolla California USA; ^6^ National Center of Neurology and Psychiatry. National Institute of Neuroscience Department of Degenerative Neurological Diseases Kodaira, Tokyo Japan; ^7^ Center for International Education and Research Tokyo Medical University Tokyo Japan; ^8^ Department of Psychological & Brain Sciences Johns Hopkins University Baltimore Maryland USA; ^9^ Graduate School of Medicine Department of Neurotherapeutics Osaka University Suita Osaka Japan; ^10^ Faculty of Medicine Department of Neurology and Life Science Research Institute Kindai University Sakai Osaka Japan

**Keywords:** amyloidogenesis, CPEB3, memory consolidation, post‐traumatic stress disorder, QBP1 transgenic mouse

## Abstract

Post‐traumatic stress disorder (PTSD) is a mental health disorder triggered by exposure to a traumatic event, which manifests with anguish, intrusive memories, and negative mood changes. So far, there is no efficient treatment for PTSD other than symptomatic palliative care. However, the cytoplasmic polyadenylation element binding protein‐3 (CPEB3) has been recently associated with a potential risk gene for PTSD. Considering that the CPEB3 protein is a functional amyloid whose importance for long‐term memory consolidation in mammals is well established, we propose the active (amyloid) state of CPEB3 as a promising potential therapeutic target to investigate the blockade of the consolidation of traumatic memories using the anti‐amyloidogenic polyglutamine binding peptide 1 (QBP1). Here we report a genetic preclinical development evaluation in mice of this concept, providing a framework for future pharmacological treatment for PTSD based on the action of the QBP1 peptide. We first characterized the human CPEB3 (hCPEB3) protein in vitro, showing how its amyloid aggregation is inhibited by the active core of QBP1 (QBP1‐M8) without affecting other self‐assembly processes such as phase separation. Then, we generated and characterized a novel transgenic mouse that constitutively expresses QBP1 in tandem (TgQBP1). TgQBP1 mice exhibit impaired consolidation of simple learning after 24 h, affecting both hippocampus‐dependent and aversive memories. While recall of the fear context is reduced—resulting in a lack of extinction—QBP1 does not affect short‐term memory. Moreover, under a PTSD‐like protocol, TgQBP1 mice showed significant extinction of the traumatic memory and reduced anhedonia compared to WT mice, which showed the expected PTSD‐like symptoms of persistent aversive memory and anhedonia. Finally, the analysis of CPEB3 amyloid presence in extracted hippocampal samples showed a correlative decrease in murine CPEB3 oligomerization in the TgQBP1 mice. Taken together, these results strongly suggest that the amyloidogenic blockade of CPEB3 by the QBP1 peptide is a potential avenue for exploring future therapeutic approaches for the prevention and treatment of PTSD, pending validation through post‐developmental or pharmacological approaches.

## Graphical abstract

Functional amyloid conversion of the translation regulator CPEB3 is essential for long‐term memory persistence. This study demonstrates that targeting this molecular switch with the anti‐amyloidogenic peptide QBP1 selectively blocks CPEB3 fibrillation without disrupting phase separation. In vivo expression of QBP1 effectively blocks memory consolidation and prevents PTSD‐like features, offering a novel therapeutic approach for traumatic stress disorders.

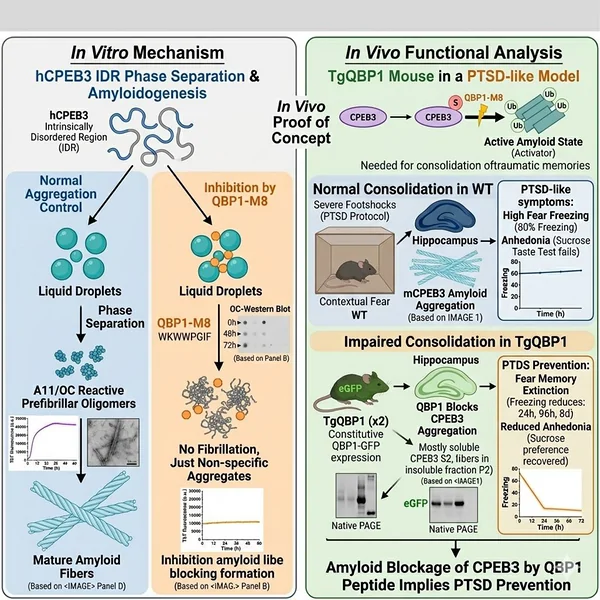

## 1 Introduction

Post‐traumatic stress disorder (PTSD) is a mental disorder classified within the group of disorders related to stress factors [1], in which exposure to a traumatic event arises specific symptoms (intrusive memories, impulsive behaviors, hyperexcitation, anxiety, and mood changes) that result in a serious alteration of the patient's life [2, 3, 4, 5]. Within the first days and weeks after the trauma, acute stress disorder (ASD) can develop as a first disturbance of the stress response [6, 7, 8], but in an unfortunate 5–8% of the general population (which is increased in war veterans, firefighters, and related jobs) these symptoms persist for more than 6 months and finally develop PTSD [9, 10, 11]. In terms of gender, the impact ratio is 1:20 in men and 1:10 in women. The factors that influence the probability of suffering this disorder are the intensity, duration and proximity of exposure to the traumatic event [12, 13, 14].

During the last four decades, most of the pharmacology for this disorder was derived from drugs already on the market for diseases such as anxiety disorders and depression [3, 15, 16]. Only selective serotonin reuptake inhibitors (SSRIs), such as Sertraline, Paroxetine, and Fluoxetine, demonstrated efficacy in the treatment of PTSD [17, 18]. However, they failed to achieve complete recovery of patients and were mainly applied to alleviate symptoms in combination with psychotherapy [14, 19]. Additionally, the use of other antidepressants such as Mirtazapine and Venlafaxine was introduced, which showed similar efficacy to SSRIs [17, 20, 21] although the corresponding clinical trials were limited due to the heterogeneity of PTSD and the complexity of its symptoms [22]. On the other hand, psychological interventions still remain as the most effective PTSD treatment, specifically cognitive behavioral therapy (CBT) coupled with focused‐trauma approaches [16, 23, 24]. Related to this, in recent years memory itself has been raised as a therapeutic target toward a specific treatment for PTSD [16, 25, 26, 27], since the primary cause of the disorder lies in the prolonged stress caused by a traumatic event and how the recall of this memory is encoded as a context (location, smell, sound, etc.) associated with a strong negative emotional valence (feelings of pain, fear, and anguish) [27, 28, 29, 30]. As a result, once the disorder has been developed, any stimulus from the environment could induce an unconscious recall of traumatic events in a safe context [3, 28, 31], triggering a crisis. Modulating those stimuli and emotional cues during active trauma recall was the main focus of these new therapies [32, 33, 34], acting specifically on fear conditioning and extinction of traumatic events. Currently, the most promising preventive pharmacology is based on the hypothalamic–pituitary–adrenal (HPA) axis and *β*‐adrenergic blockers [16, 35]; thus, hydrocortisone [25, 36, 37], oxytocin [38, 39, 40], and propranolol [26, 41, 42, 43] are able to reduce the impact of fear memories (in human and rodents) and ameliorate PTSD symptoms, but they are still more focused on reconsolidation‐based approaches in combination with psychological interventions [23, 25, 26, 42, 44] than being a first‐line solution itself.

Based on this approach, one could expect that directly interfering on protein synthesis and synaptic plasticity would be a more effective strategy, as it will directly affect the very first consolidation process after the trauma exposure. In the search for new specific therapeutic targets related to memory consolidation and PTSD onset, the CPEB family stands out as the most promising one since they are RNA‐binding proteins capable of regulating mRNA translation and mediate memory storage and synaptic plasticity [45, 46, 47], along which CPEB3 isoform is considered as a potential risk gene for PTSD [48]. In vertebrates, CPEB is usually coded by four genes of which the first member identified was CPEB1, with CPEBs 2–4 later described in mice [49, 50, 51, 52]. This family of proteins share a RNA‐binding domain (RBD) at their C‐terminal regions and in the case of CPEB2 and CPEB3 there are N‐terminal regions rich in glutamine/asparagine residues (Q/N regions), which resemble the amyloid/prionoid Q/N region of their orthologues in *Aplysia* and *Drosophila* (*Ap*CPEB and Orb2, respectively) [49, 53, 54, 55]. CPEB3 is able to form self‐propagating and heritable aggregates in yeast and its aggregation in mice brain occurs only under physiological conditions after neuronal stimulation, associated with long‐term memory (LTM) [54, 56]. Furthermore, CPEB3 knockout mice (KO) showed defects in memory consolidation associated with spatial learning and the interruption in CPEB3 function after consolidation result in an impairment in long‐term memory [56, 57, 58]. CPEB2 is also required for long‐term memory consolidation [59, 60], but its amyloidogenic and prionoid properties have not been characterized yet. Considering the functional importance of the Q/N prionoid regions of the CPEB family, their evolutionary conservation [45, 50] and their function as a switch for long‐term memory through protein synthesis in mammals, CPEB3 constitutes a promising therapeutic target for pharmacological interventions for PTSD.

Polyglutamine binding peptide 1 (QBP1: SNWKWWPGIFD) is an anti‐amyloidogenic dodecapeptide originally developed to specifically bind to long polyQ stretches (i.e., pathological) [61, 62], as a therapeutic approach for neurodegenerative diseases known as polyglutaminopathies [63, 64]. According to the proposed mechanism of action, QBP1 specifically binds to the protein monomer and is capable of blocking the transition of the toxic β‐sheet rich conformation that initiates amyloidogenesis [65, 66], thus blocking this process at its start. In addition to polyQ regions, QBP1 has shown polyvalence by blocking the conformational polymorphism of polyQ, Sup35NM, and α‐synuclein at the monomer level [65, 67], as well the amyloidogenesis process of TDP‐43 [68]. Interestingly, it was also demonstrated that the minimum active core named QBP1‐M8 (octapeptide: WKWWPGIF) [69, 70] also inhibits in vitro the amyloidogenesis of functional amyloids like *Ap*CPEB [71] and Orb2 [72]. Also, a preliminary report on the action of QBP1 on CPEB3 was recently advanced [73]. Moreover, the expression of a tandem QBP1 construct in a *Drosophila* transgenic model showed that it is able to block memory consolidation and learning [72]. Based on all this, we proposed that the glutamine based structures present in murine CPEB3 could also be blocked by QBP1, allowing us to explore its feasibility to develop a specific pharmacological approach for PTSD.

Here, we have first characterized the interplay between QBP1 and CPEB3 protein in vitro, confirming the inhibition of the amyloid state and analyzing the nature of their interaction. Second, we have generated and characterized a transgenic mouse constitutively expressing QBP1 in tandem (TgQBP1 mice), showing an impairment in long‐term memory accompanied by a decreased mCPEB3 aggregation at hippocampal samples in the presence of QBP1. Our results provide a proof of concept of a novel and specific genetic strategy targeting memory consolidation of traumatic memories in mammals by QBP1, which could serve as a baseline for developing future therapies for preventing and treating PTSD and ASD.

## 2 Results

### 2.1 Comprehensive Characterization of QBP1 Inhibition of Human CPEB3 Amyloidogenesis

The effect of QBP1 on human CPEB3 amyloidogenesis was recently advanced in our previous work [73], where QBP1 was used primarily as an amyloid modulator. Here we have fully characterized its inhibitory effects on amyloidogenesis. We first monitored the amyloid aggregation kinetics of the hCPEB3 intrinsically disordered region (hIDR_1–450_) [73] using Thioflavin T (ThT) fluorescence intensity, an amyloid stain that binds to the structure of the mature amyloid and increases its fluorescence emission at 490 nm [74]. Moreover, we have examined amyloid kinetics in the presence of either the QBP1 active core (QBP1‐M8, WKWWPGIF) or its *Scrambled* version (SCR‐C: WPIWKGWF) as a permutation control [62, 69, 70]. Amyloid kinetics in the presence of QBP1‐M8 and SCR‐C at a ratio of 1:8 (protein:inhibitor) showed that both peptides were able to inhibit hIDR fibrillation (Figure 1A). We further analyzed whether QBP1‐M8 inhibition affects the different intermediate amyloid species formed by hIDR by immunodot blot and SDD‐AGE probed with A11/OC conformational antibodies [75], which specifically interact with the native conformation of prefibrillar oligomers (often associated with toxic species, A11 antibody) or fibrillar oligomers and mature fibers (OC antibody). The immunodot blot analysis of hIDR, performed with light agitation to minimize unspecific aggregation, has shown an inhibition of the formation of both prefibrilar oligomers and mature oligomers and fibers in the samples containing QBP1‐M8 (Figure 1B). The SDD‐AGE analysis also showed a reduction in the formation of aggregates/oligomers smears in the presence of QBP1‐M8 (Figure 1C). The species formed throughout the aggregation cascade were then visualized in the presence of QBP1‐M8. In the TEM micrographs of hIDR we observed the formation of non‐specific aggregates mixed with some fibrils even at 216 h when QBP1‐M8 is present (Figure 1D), with a reduction of fiber formation indicative of the inhibition of its amyloidogenesis. In the case of the amyloid forming region [73], named hCPEB3 R1 (residues 1–200, Figure S1A), we observed enhanced non‐specific aggregation and fibril‐like assemblies still present, which is consistent with an enhancement of its aggregation kinetics in the presence of QBP1‐M8 (Figure 1E). Moreover, a longer amyloid region of the hCPEB3 IDR (named R2 [73], spanning 1–300 residues) showed abundant fibrillation *in vitro*, which in the presence of QBP1‐M8 showed a large amount of protofibrils evolving into amyloid fibers mixed with non‐specific aggregates (Figure S1B). Taken together, these results suggest that QBP1 inhibits the amyloidogenesis of the IDR and hCPEB3 regions (kinetics as well as fiber and oligomer formation), which is not complete because there is some residual mature amyloid formation in the sample (especially depending on the particular hCPEB3 region analyzed).

**FIGURE 1:**
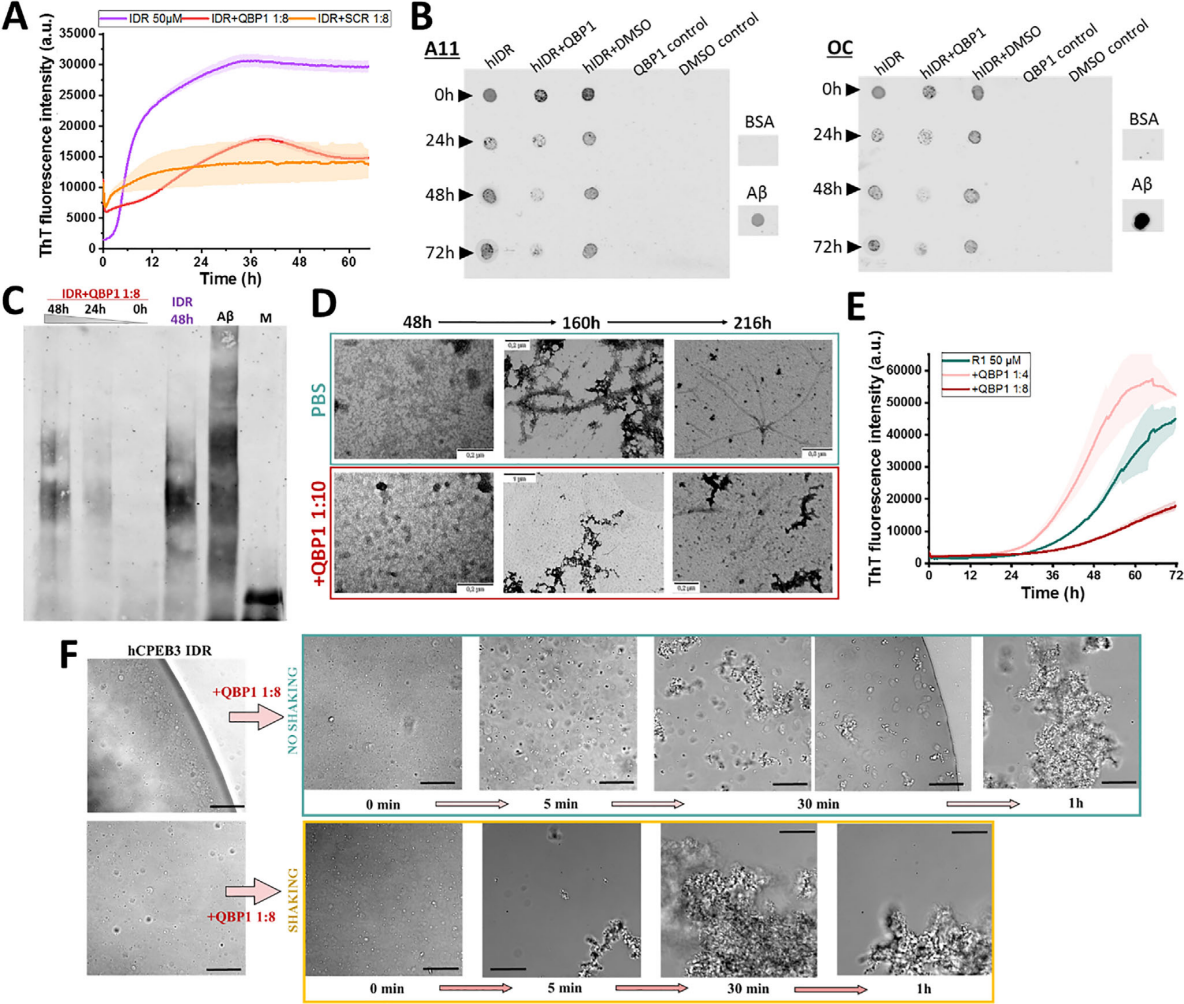
QBP1‐M8 inhibits the amyloidogenesis of hCPEB3 IDR and its fragments. (A) Kinetics of amyloid aggregation of hCPEB3 IDR (50 µM) monitored by ThT fluorescence emission in the presence or absence of QBP1‐M8 (1:8 ratio) and SCR‐C (1:8 ratio). Error bars indicate the standard error of the mean (*n* = 3) of a representative experiment. (B) Immunodot blot of hCPEB3 IDR (50 µM, purple) in the presence or absence of QBP1‐M8 (1:8, red) or DMSO (vehicle, 1:8, yellow), which reflects the presence of A11/OC reactive species formed after incubation at 37 °C with shaking (800 rpm thermoblock). Aβ and BSA were used as positive and negative controls, respectively. (C) SDD‐AGE of hCPEB3 IDR (40 µM) in the presence or absence of QBP1 (1:8) at different incubation time points at 37 °C with shaking (800 rpm, thermoblock) and immunoblotted with an anti‐OC antibody (against fibrillar oligomers and fibers). Aβ was used as a positive control. (D) Representative images of hIDR (10 µM) fibrillation in the presence or absence of QBP1‐M8 (1:10). Samples were incubated at 37 °C in PBS pH 7.4 without shaking. Scale bars are indicated for each sample. (E) Kinetics of hCPEB3 R1_1–200_ (50 µM) amyloid aggregation, monitored by ThT fluorescence emission, in the presence or absence of QBP1‐M8 (1:4–1:8). Error bars indicate the standard error of the mean (*n* = 3) of a representative experiment. (F) Phase‐contrast micrographs of the effect of QBP1‐M8 (1:8 ratio) on the phase separation of hCPEB3 IDR (50 µM) upon co‐incubation in the absence (upper turquoise panel) or presence of agitation (37 °C and 800 rpm, lower yellow panel) under physiological relevant conditions (PBS pH 7.4). Scale bar: 0.5 µm.

Finally, we examined whether QBP1‐M8 peptide affects the phase separation behavior of hCPEB3 IDR [73]. The addition of QBP1‐M8 (1:8 ratio) to liquid droplets of this protein incubated without agitation showed that phase separation is maintained over time until formation of aggregates saturates the protein concentration (Figure 1F, upper turquoise panel). Upon agitated co‐incubation, nonspecific aggregates formed earlier (Figure 1F, lower yellow panel). These results suggest that QBP1‐M8 lowers amyloid aggregation without altering the phase separation process.

### 2.2 QBP1 Transgenic Mice Showed Impaired Long‐Term Memory

Once we demonstrated *in chemico* that QBP1‐M8 inhibits the active amyloid state of CPEB3, we set out to test the in vivo proof of concept that QBP1 could maintain its inhibitory effect and in turn block memory consolidation in a mammalian model. To that end, we generated a transgenic mouse constitutively expressing QBP1 in tandem (SNWKWWPGIFD‐SNWKWWPGIFD), to further increase its activity [66, 76], fused to the green fluorescence protein (eGFP) (TgQBP1, Figure S2A,B). After verifying the presence of QBP1 in the hippocampus of the TgQBP1 mice (Figure S2C), we performed an extensive battery of general health and developmental tests in order to discard gross defects (Figure S2D).

First, we characterized the locomotor activity of TgQBP1 mice by analyzing them in a two‐day protocol (Figure 2A): while Day 1 measures the locomotor activity itself, where the animal freely explores the apparatus cage, its re‐exposure on Day 2 (24 h later) reflects their exploratory behavior in a familiar environment (usually lower). The horizontal activity showed normal locomotor activity with no differences compared to control mice (WT) on Day 1 (Figure 2B), although TgQBP1 mice were significantly more active than WT mice on Day 2 (*p* < 0.001), even if both groups significantly decreased their activity compared to the previous day (WT *p* < 0.001 and TgQBP1 *p* = 0.017). This behavior was similar for the vertical activity (Figure S3A), where TgQBP1 mice significantly displayed an increased activity on Day 2 (*p* = 0.027). Indeed, the horizontal activity by minute evinced that mice varied their activity according to the minute, day and group (*p* = 0.028; Figure 2C). However, on Day 1 mice decreased the activity over time regardless of group (*p* < 0.001), but on Day 2 TgQBP1 mice had significantly higher overall activity than WT mice (*p* < 0.001) and specifically in the last minutes of the test (Min3 *p* < 0.001; Min4 *p* = 0.003; Min5 *p* = 0.01). Then, to examine whether this effect relied on different basal levels of anxiety, we performed an Elevated plus maze test. We did not find significant differences in any of the analyzed variables (Figure S3B,C), which suggests normal levels of anxiety in the TgQBP1 mice. Taken together, we propose that abnormal re‐exploratory behavior of the TgQBP1 mice could originate from an impairment in the memory consolidation of the known environment.

**FIGURE 2:**
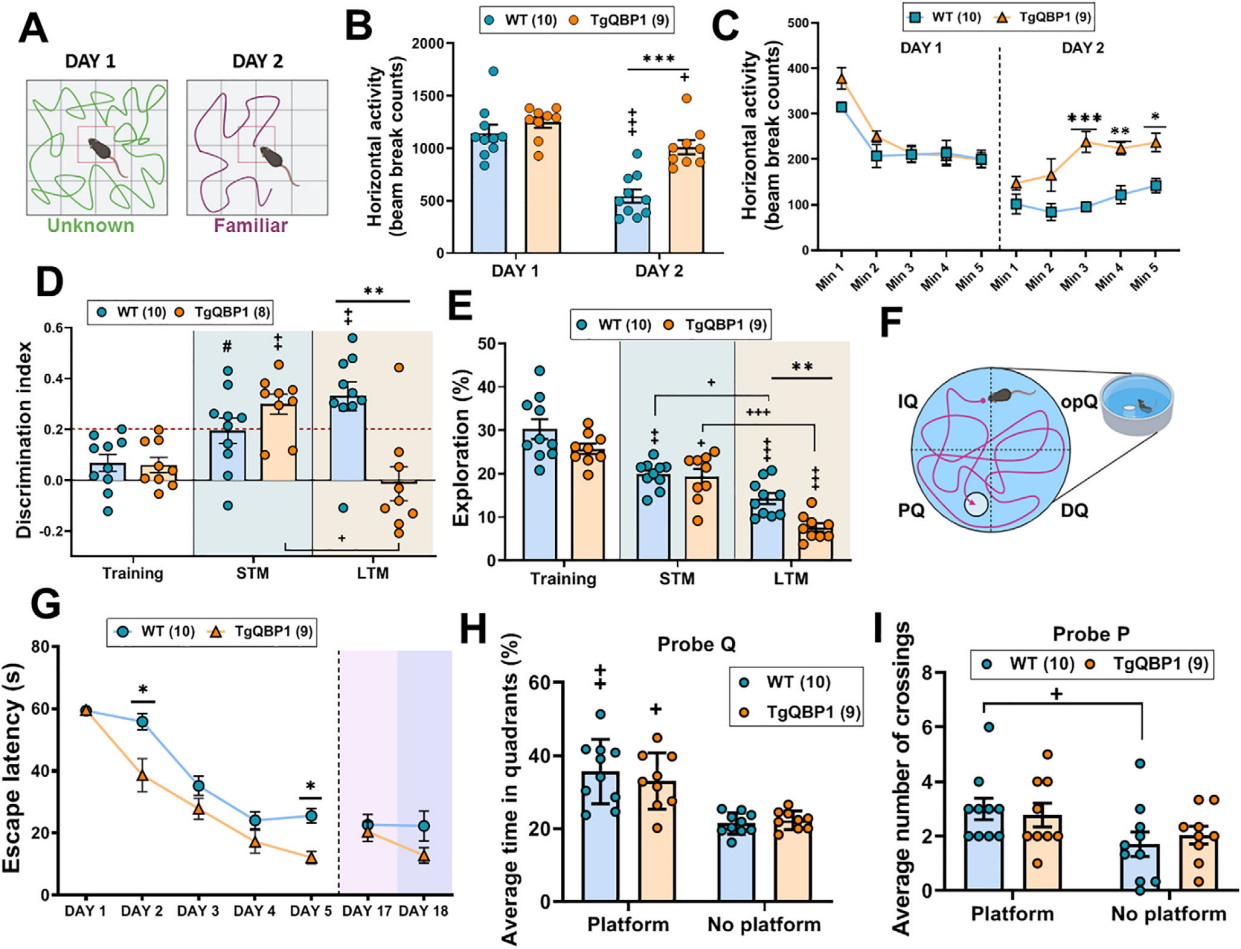
Hippocampal‐related memory is impaired in the TgQBP1 mice. (A) Representative schematic of the two‐day activity cage protocol: on the first day, the locomotor activity is assessed in a novel environment, whereas on the second day the locomotor activity is analyzed in a familiar environment. (B) Total horizontal activity on both days of the protocol in the activity cage. On Day 1, no differences between groups were observed, whereas re‐exposure on Day 2 showed that TgQBP1 mice significantly increased their activity compared to WT mice (*p* < 0.001). Furthermore, both groups significantly decreased their activity compared to Day 1 (WT *p* < 0.001 and TgQBP1 *p* = 0.017). (C) The horizontal activity per minute on each test day reflected that mice modify their behavior as a function of day, minute and group (Interaction *p* = 0.028). On Day 1, there were no significant differences between groups, although both groups changed their behavior significantly over minutes (Time *p* < 0.001). On Day 2, there was no interaction, but both groups varied their behavior over time (*p* = 0.0014) and as a function of group (*p* < 0.001). Specifically, TgQBP1 mice showed significantly more activity than WT mice in the last minutes of the test (*p* < 0.001, *p* = 0.003, and *p* = 0.01 respectively). (D) Discrimination Index (DI) values are shown for each phase of the test. WT mice tended to show higher DI scores in the STM phase (*p* = 0.059) and significantly higher DI scores in the long‐term monitoring (LTM) phase (*p* = 0.007). TgQBP1 mice showed significantly higher DI scores in the short‐term memory (STM) phase compared to the training phase (*p* = 0.01), but showed significantly lower DI scores in the LTM phase (*p* = 0.025) and significantly lowered their DI scores in the LTM phase compared to the STM phase (*p* = 0.012). Finally, TgQBP1 mice tended to show higher DI scores than WT animals in the STM phase (*p* = 0.057) and significantly lower DI scores in the LTM phase (*p* = 0.002). The red dotted line corresponds to the discrimination threshold (0.2). (E) The exploration percentage reflects that there is a significant interaction between the time of the test and the group (*p* = 0.049). WT mice significantly decreased their exploration with respect to training (STM *p* = 0.002 and LTM *p* < 0.001), as well as significantly decreased it from short to long term (*p* = 0.018). TgQBP1 mice also significantly decreased their exploration compared to training (STM *p* = 0.016 and LTM *p* < 0.001) and between short and long term (*p* < 0.001). However, notably TgQBP1 mice explored the novel object significantly less in the long term (*p* = 0.002). (F) Representative diagram of the Morris water maze. This test consists of evaluating the spatial and cognitive memory of animals, which must find a transparent platform located in one of the four quadrants of a pool (PQ). For several days, four times each day, the mice learn the location of the platform and take increasingly less time to reach it (escape latency). (G) Acquisition of platform localization, where escape latency decreased over days in both groups, especially in the TgQBP1 mice (Day 2 *p* < 0.001 and Day 5 *p* < 0.001). However, LTM at 17–18 days reflected a loss of that improvement (pink‐lilac). (H) Percentage of time in each quadrant after acquisition (Probe Q), in which both groups spent significantly more time in PQ than in the average of the other quadrants (WT *p* = 0.004 and TgQBP1 *p* = 0.014). (I) Mean number of crossings over the platform location (Probe P), in which only WT mice significantly crossed the exact location of the platform in the PQ quadrant more times than TgQBP1 mice with respect to the same location in the other quadrants (*p* = 0.017). For comparisons between independent groups: *p < 0.05, **p < 0.01, ***p < 0.001, trends 0.05 ≥ # < 0.09; for comparisons between dependent groups: +*p* < 0.05, ++*p* < 0.01, +++*p *< 0.001, trends 0.05 ≥ #′ < 0.09.

Next, we analyzed the integrity of the hippocampus‐dependent memories in TgQBP1 mice. A novel object recognition test was performed to evaluate the ability of rodents to recognize a novel object in the absence of reinforcement (Figure S3D), where they should spend more time exploring the novel object as they have a natural preference for novelty [77]. According to the discrimination index (DI, exploration of the new object compared to total exploration), both groups equally explored the two unfamiliar objects in the training phase (Figure 2D). One hour later (short term, Figure 2D turquoise), the two groups explored the novel object significantly more according to training (WT *p* = 0.089 and TgQBP1 *p* = 0.004). After 24 h (long term, Figure 2D beige), WT mice improved their discrimination in comparison to training (*p* = 0.002), while TgQBP1 mice significantly worsened it in comparison to short term (*p* = 0.041), showing a significant difference between the two groups (*p* = 0.003). Overall, TgQBP1 mice were able to discriminate novelty at short term (1 h) but their performance was completely deteriorated at long term (24 h), exploring both objects equally as if they were completely unfamiliar (Figure 2D). In terms of exploration (number of times the animal approaches the object frontally), the percentage reflected that there were significant differences depending on the group and test phase (*p* = 0.049; Figure 2E), and they also needed fewer explorations to discriminate the novel object (*p* < 0.001). However, TgQBP1 mice explored significantly less than WT mice at long term (*p* = 0.002), suggesting that their lack of discrimination causes them to spend more time within each exploration. Finally, the percentage of novelty preference (Figure S3E), in which a 50% of exploration means an equal interest in both objects (purple dashed line), confirmed that TgQBP1 mice did not significantly discriminate the novelty after 24 h (long term, *p* = 0.003), confirming the memory impairment at the long‐term phase.

### 2.3 TgQBP1 Mice Showed an Impaired Memory Consolidation Despite Repetitive Learning

Considering that the function CPEB3 is based on its conversion to an active amyloid state and that its prion‐like properties promote temporal stability, we decided to assess memory consolidation in a repetitive and more complex learning paradigm. The Morris water maze is a hippocampal‐dependent task widely used to study spatial learning and memory in rodents, in which mice learn to escape from the pool by finding a transparent platform along four trials per day (Figure 2F). In the acquisition phase, both groups significantly decreased their escape latency over days (Days *p* < 0.001), showing that they had learned the task (Figure 2G). Surprisingly, TgQBP1 mice seemed to performe significantly better than WT mice over time (Group *p* = 0.001) and they performed significantly better on the last day of acquisition (Day 5, *p* < 0.001) (Figure 2G), likely because WT mice performance reached a *plateau* on that final day.

After the acquisition, we analyzed the recall of the platform location. Specifically, we analyzed the time spent in each quadrant (Probe Q, from *quadrant*), where both groups spent significantly more time in the quadrant with the platform (PQ, WT *p* = 0.004 and TgQBP1 *p* = 0.014) (Figure 2H). However, when we analyzed the number of times they have crossed the platform location in that quadrant at the millimeter level (Probe P, from *platform*), only WT mice spent significantly more time on the platform than off the platform (*p* = 0.017; Figure 2I). Taken together, these results suggest that TgQBP1 mice remember the quadrant of the platform, but not the position of the platform within the quadrant, which evidences some impairment of memory consolidation. We then analyzed their long‐term memory by exposure to the task 12 days later (Day 17, pink), observing that the TgQBP1 mice increased their escape latency compared to Day 5 (*p* = 0.047) while the WT mice maintained a similar latency (Figure 3A). Although there were no differences between groups and both groups learned and memorized the task, it should be noted that TgQBP1 mice seem to remember the platform location worse at the long term, which could rely on an impairment in memory consolidation. In fact, another maze exposure on the following day at the long‐term revision (Day 18—lilac, Figure 3B) showed a reduction in the escape latency of TgQBP1 mice in relation to the previous day (*p* = 0.043), suggesting that TgQBP1 mice were then able to remember the platform. Together, these results suggest that TgQBP1 mice retain some memory (localization of the quadrant on Probe Q and decreased latency on Day 18), but have impaired long‐term memory (failure to localize the platform on Probe P and increased latency on Day 17).

**FIGURE 3:**
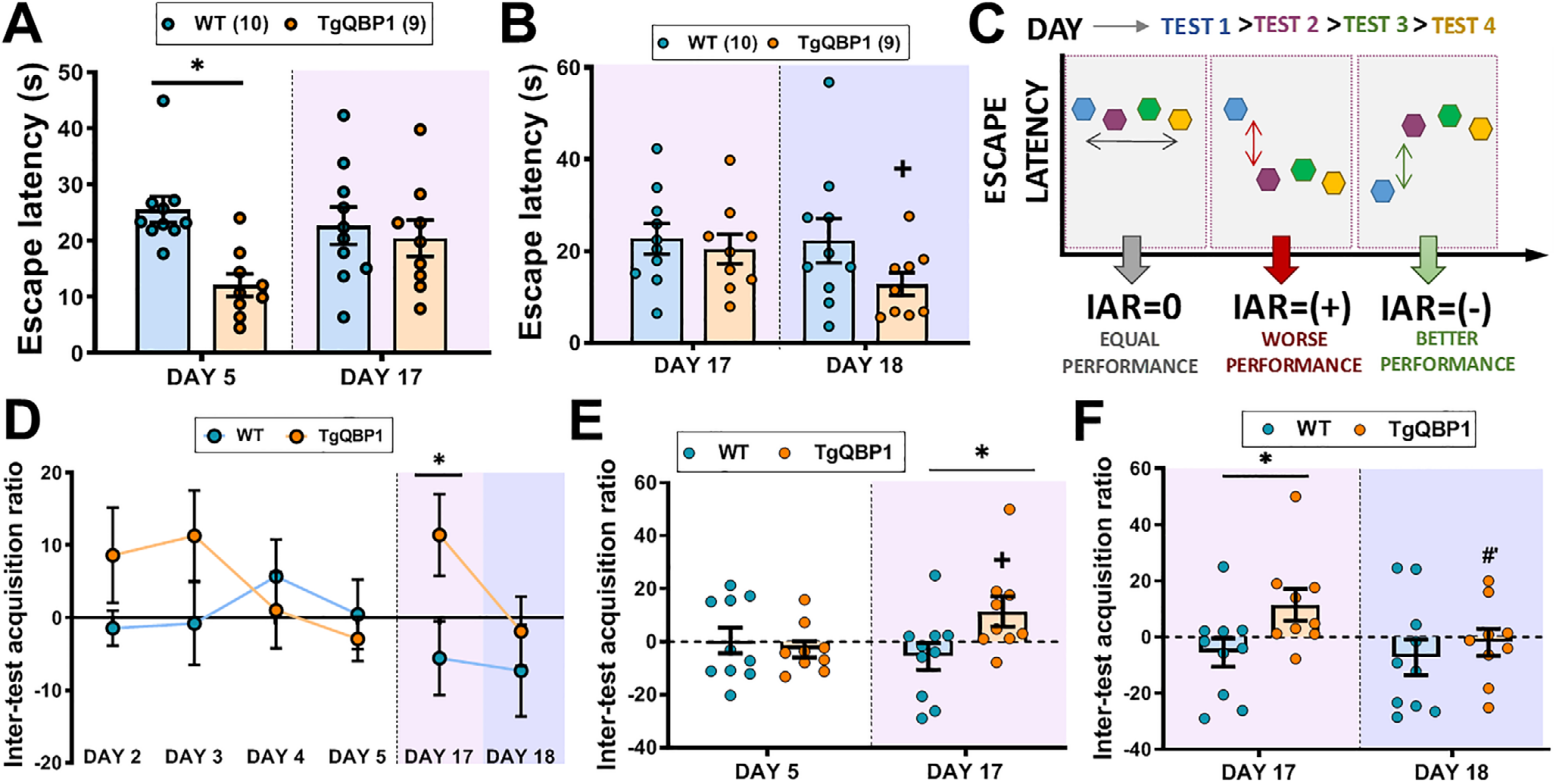
TgQBP1 mice showed intra‐subject memory impairment in acquisition trials. (A) Comparison of escape latency between Day 5 of acquisition and Day 17 (LTM, pink), showing a loss of significant difference between the last day of acquisition and the long‐term test in TgQBP1 mice (*p* = 0.047). (B) Comparison of escape latency between Day 17 and Day 18 (LTM, lilac), showing an improvement of escape latency in TgQBP1 mice 24 h after LTM testing (*p* = 0.043). (C) Inter‐trial acquisition ratio (IAR) of the Morris water maze, which reflects the animal's performance on the first trial (Trial 1) of each day of task acquisition. This ratio is obtained by subtracting the mean of the four trials performed each day of acquisition from the escape latency of Trial 1. If the animal's performance on Trial 1 is similar to the day's mean (left panel), the IAR will have a value close to zero. On the other hand, if the animal's performance is worse (middle panel), the IAR will be positive (higher escape latency in Trial 1); whereas if the animal's performance is better than the day's mean (right panel), the IAR will be a negative value (lower escape latency in Trial 1). (D) Representation of the inter‐trial acquisition ratio (IAR) of WT mice (turquoise) relative to TgQBP1 mice (orange). The IAR of WT mice is close to zero during the first days of acquisition (equal performance in all trials) and then decreases as the mice remember the platform location (better performance). In the TgQBP1 mice, the IAR is highest on the first days of acquisition (worse performance on Trial 1 of each day) and then starts to decrease until day 5 (better performance because they remember the platform). However, on day 17 it increases significantly (*p* = 0.039; worse performance) and 24 h later it decreases again (better performance after re‐exposure to the test). (E) Comparison of the IAR between day 5 of acquisition and day 17 reflects that the TgQBP1 mice significantly worsened their performance on the first trial on day 17 (*p* = 0.042) with respect to the last day of acquisition. A significant difference between groups on Day 17 is also shown (*p* = 0.039). (F) Comparison of the IAR between Day 17 and Day 18 reflects that the TgQBP1 mice showed a trend toward improving their performance on Trial 1 on Day 18 with respect to Day 17 (*p* = 0.091), reaching similar values to WT mice; For comparisons between independent groups: *p < 0.05, **p < 0.01, ***p < 0.001, trends 0.05 ≥ # < 0.09; for comparisons between dependent groups: +*p* < 0.05, ++*p* < 0.01, +++*p* < 0.001, trends 0.05 ≥ #′ < 0.09.

To analyze this variation in performance, we analyzed whether the impaired consolidation of TgQBP1 mice also occurs within each day of acquisition (24 h). Plotting the escape latency of the first trial (Trial 1) against the last trial of the day (Trial 4) showed that TgQBP1 mice significantly varied its performance along each trial within the same day (Figure S3F), specifically a trend on Day 5 (*p* = 0.063) and a significant variation on Day 17 (*p* = 0.040). In contrast, the performance of WT mice was homogeneous across all trials within a single day (Figure S3G). We have defined a new variable we called Inter‐trial Acquisition Ratio (IAR) to reflect the performance of the mice in Trial 1 in comparison to the mean of the four trials of each acquisition day (Figure 3C). IAR values for both groups reflected that WT mice had an IAR close to zero on the first days of acquisition (Figure 3D, blue), which correlates with their performance being consistent across all trials on the same day. In contrast, TgQBP1 mice had very high IAR values along the first days of acquisition, suggesting a worse performance in Trial 1 compared to the other trials within a day (Figure 3D, orange). In both groups, IAR value decreased from Day 4 onwards as they remembered the task and improved their performance on Trial 1. Finally, comparison of IAR values between acquisition (Day 5) and long‐term re‐exposure (Day 17, pink) reflected a significant increase in the IAR value in the TgQBP1 mice relative to WT (*p* = 0.039; Figure 3E) and relative to their own performance on acquisition (*p* = 0.042), again suggesting a worse performance after non‐exposure to the task. However, this underperformance was recovered on Day 18 (lilac, Figure 3F), where the IAR value of TgQBP1 mice was almost zero due to a tendency to improve their performance (*p* = 0.091). Overall, the IAR variable has highlighted the impairment of long‐term memory between each test day (every 24 h), evinced by a worse performance on Trial 1 that improves with subsequent trial exposures within the same day. We suggest that, although QBP1 is inhibiting memory consolidation, the learning of this task is repetitive (4 trial/day) and extended in time (3–5 days) so that memory consolidation seems to be good enough for the mice to recall the task after a single re‐exposure (better performance on Day 18).

### 2.4 QBP1 Impairs Long‐Term Consolidation of Aversive Memories

After demonstrating that QBP1 was capable of impairing long‐term consolidation of hippocampal‐dependent memories, we proceeded to analyze aversive memories. To do this, we used the contextual fear conditioning test, a paradigm of associative memory where the aim is to condition a traumatic event (small, inescapable shock; unconditioned stimulus—UCS) to a context and an acoustic cue (conditioned stimulus, CS) [78], following the experimental design described in Figure 4A. WT and TgQBP1 mice correctly acquired the aversive conditioning, which is reflected in the significant increase in the freezing percentage along each trial within a session (Trial *p* < 0.001, Figure 4B). After 24 h, recall of the fear context was analyzed by showing the freezing percentage per minute (Figure 4C), where there was an interaction effect (*p* = 0.008), based on both groups varying their behavior over time (Min *p* < 0.001) and that TgQBP1 mice showed significantly less freezing than WT mice (Group *p* = 0.046), suggesting impaired aversive memory in TgQBP1 mice.

**FIGURE 4:**
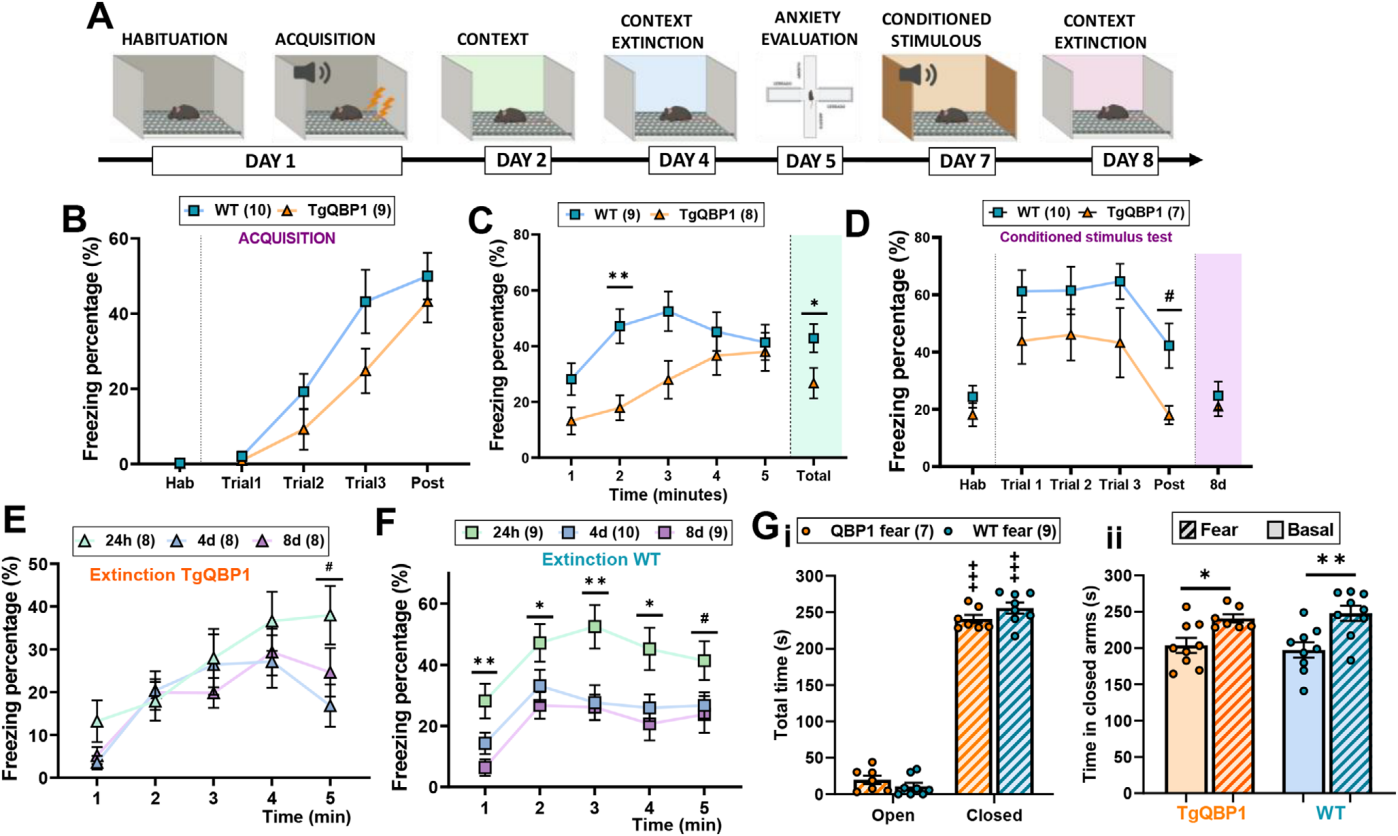
Analysis of aversive memories in TgQBP1 mice. (A) Schematic of the experimental design for the contextual fear conditioning test. The diagram shows the temporal sequence of the test implemented in the TgQBP1 mice for fear conditioning establishment. (B) Percentage of fear in the acquisition phase of fear conditioning, showing an increase in percentage with trials of the test for the two groups, with no significant differences between them. (C) The percentage of fear in the context reviewed at 24 h after acquisition reflects that there are significant differences according to group and minute of the test (Interaction *p* = 0.008). The TgQBP1 mice show a significant overall lower context fear behavior than WT (Group *p* = 0.046), which broken down by minute shows a significant difference between groups at Min 2 (*p* = 0.009). In addition, both groups show an overall increase in fear over test minutes (Time *p* < 0.001). In terms of total time (green), the TgQBP1 mice showed significantly less freezing than the WT (*p* = 0.046). (D) The conditioned stimulus test (day 7) analyzes how the animal remembers the acoustic tone conditioned to the aversive event (shocks) in a different context (non‐aversive). Both groups modify their behavior across trials (*p* ≤ 0.001) and WT mice show a tendency to exhibit a higher percentage of global fear (Group *p* = 0.051). This means that WT mice remember the conditioned stimulus, with a significant trend in the post‐test phase with respect to the TgQBP1 mice (*p* = 0.057). However, there are no differences in the total freezing percentage in the context review 24 h after (8 d). (E) Intra‐subject analysis of fear extinction per minute for the TgQBP1 mice. There are no differences in their overall behavior between the three days of context evaluation but they change over the minutes of the test (24 h *p* < 0.001, 4 d *p* < 0.001 and 8 d *p* = 0.03). Among extinction times, the TgQBP1 mice only tend to change their behavior at Min 5 (*p* = 0.061). (F) Intra‐subject analysis of fear extinction per minute for WT mice. This group shows that animals change their behavior over the test minutes (*p* < 0.001) and globally extinguish conditioning to the context (*p* = 0.002), especially from 24 h to 4 d–8 d (*p* < 0.001). Furthermore, because of this, WT mice show different behavior per minute between days of exposure to context: Min 1 (*p* = 0.005), Min 2 (0.044), Min 3 (*p* = 0.008), Min 4 (*p* = 0.016) and Min 5 (*p* = 0.090). (G) Results from the elevated plus maze to compare anxiety levels in basal versus aversive conditions (fear). i) In the fear condition, the total time spent in the arms showed that both groups and in all conditions spent significantly more time in the closed arms (*p* < 0.001). ii) In the comparison of basal versus fear conditions in total time in closed arms, the analysis revealed that both groups increased their time after fear conditioning (WT *p* = 0.003 and TgQBP1 *p* = 0.038). For comparisons between independent groups: *p < 0.05, **p < 0.01, ***p < 0.001, trends 0.05 ≥ # < 0.09; for comparisons between dependent groups: +*p* < 0.05, ++*p* < 0.01, +++*p* < 0.001, trends 0.05 ≥ #′ < 0.09.

In the long term, the recall of the conditioned stimulus was assessed at the Cue test (acoustic cue, Figure 4D). These results showed that, overall, TgQBP1 mice tended to exhibit less fear than WT mice (Group *p* = 0.051, specifically at Post phase *p* = 0.057) in response to the conditioned stimulus, but also both groups varied their behavior over trials (*p* < 0.001) as they reduced their freezing at the end of the test (associative fear extinction). However, when we analyzed the average freezing performed by each group during Cue test phases where the acoustic cue is present (Trials, red) compared to when it is absent (ITI and Post, lilac), only control mice significantly decreased their freezing during ITI (*p* = 0.011, Figure S4A). Altogether, these results suggest that the TgQBP1 mice also showed impaired long‐term memory of the conditioned stimulus, which could be related with a reduced associative fear or a recall impairment.

Next, we analyzed the extinction over context recall for each group (intra‐subject): TgQBP1 mice showed an absence of global extinction (Extinction *p* = n.s.) although there is an interaction (*p* = 0.030) since their freezing percentage varies significantly over the minutes of testing within each day of extinction (Minutes *p* < 0.001) (Figure 4E). On the other hand, WT mice performance showed a significant extinction of the fear context (Extinction *p* = 0.002, Figure 4F), although there is some effect of time (Minutes *p* < 0.001) as they always show less freezing in Min 1 than in the other minutes of the test. Furthermore, the correlation analysis revealed that WT mice extinguish their freezing [*r* = −0.5221 *R* ^2^ = 0.2726 *p* = 0.045] while TgQBP1 mice maintain a linear behavior over time [*r* = −0.0375 *R* ^2^ = 0.0014 *p* = n.s.] (Figure S4B). We then compared the total freezing time on each aversive context test (24 h–4 d–8 d, Figure S4C), which also confirms that only WT mice significantly extinguished their freezing between extinction days [*F*_(2,24)_ = 3.975 *p* = 0.032] and showed a trend between 24 and 96 h of extinction (*p* = 0.081). Overall, TgQBP1 mice showed a lower response to conditioned fear in both context and acoustic tone. According to our working hypothesis, this behavior may be attributed to an impairment in the consolidation process of the conditioned stimulus, leading to insufficient extinction of the memory.

Finally, to assess the emotional impact we analyzed the anxiety levels on an Elevated plus maze after the fear conditioning and compared to levels in basal conditions (Figure S3B,C). Considering only the comparison in fear conditions (mice that have learned an aversive memory), both groups significantly spent more time in closed arms (*p* < 0.001, Figure 4Gi). Moreover, when we compare the total time spent in closed arms (Figure 4Gii), both groups significantly spent more time in closed arms in fear conditions (WT: *p* = 0.003/ TgQBP1: *p* = 0.038). This difference relates to the significant decrease in the exploration frequency of both groups in fear conditions (WT: *p* < 0.001/TgQBP1: *p* = 0.001) (Figure S4D), but not in the percentage of entries (Figure S4E), which is increased in closed arms for both groups in fear conditions (WT: *p* < 0.001/TgQBP1: *p* = 0.004; Figure S4Ei) and remains similar to those on basal conditions (Figure S4Eii). Taken together, these results suggest that both WT and TgQBP1 mice increase their anxiety levels after the fear conditioning, although it seems to be more pronounced in the WT mice. This may suggest that, although TgQBP1 mice exhibit impaired consolidation of the aversive memory, the emotional impact of the traumatic experience is still present and leads to increased anxiety.

### 2.5 Effects of Age and Sex on Memory Impairment in TgQBP1 Mice

After demonstrating the effect of QBP1 on memory consolidation in adult male mice, we decided to expand our study to investigate the effects of age and sex. Firstly, it has been reported that younger individuals develop PTSD more often due to their more vulnerable brains and greater exposure to high‐risk situations [10, 79]. This can significantly impact their lives for over a decade and impose a substantial economic burden on the healthcare system [79, 80]. Also, aversive memory consolidation in adolescent mice differs in extinction compared to adults, which may explain the challenges in treating anxiety disorders in younger populations [80, 81]. To analyze the effect of QBP1 in younger animals, male mice aged P90 were subjected to contextual fear conditioning (Figure 5A). During acquisition, TgQBP1 and WT groups significantly learned the task (Trial *p* < 0.001) with no differences between them. Analysis of the aversive context recall at 24–96 h indicated that both groups globally extinguished freezing behavior (Time *p* = 0.003) but showed overall differences (Group *p* = 0.054), with younger TgQBP1 mice showing a trend to perform less total freezing compared to WT mice at 24 h (*p* = 0.051). This suggests that the effect of QBP1 on impaired aversive memory consolidation is similar in younger male mice.

**FIGURE 5:**
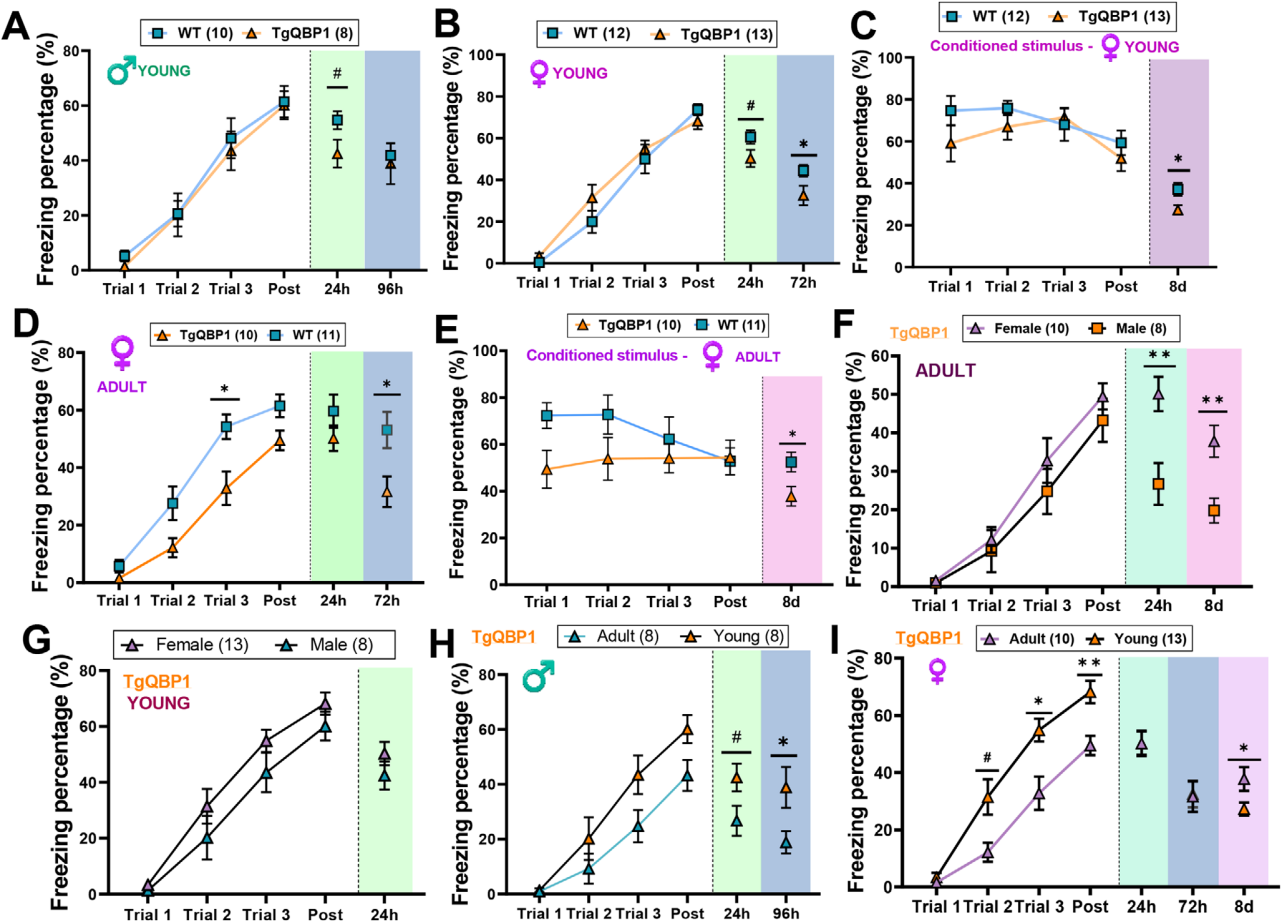
Effects of age and sex on fear conditioning in TgQBP1 mice. (A) Percentage of fear in younger male mice during aversive conditioning. During the acquisition phase, both groups learned the conditioning task significantly across trials, with no differences between them. However, at 24 h post‐training, TgQBP1 mice showed a trend toward poorer recall of the fear context compared to WT mice (*p* = 0.051). By 96 h,the significance of this difference was lost as both groups exhibited similar fear extinction. (B) Percentage of fear in younger female mice during aversive conditioning. Both groups learned the conditioning task significantly during acquisition, with no differences between them. However, at 24 h post‐training, TgQBP1 mice showed a trend toward less total fear compared to WT mice (*p* = 0.066), with this difference becoming more pronounced at 72 h (*p* = 0.044). (C) Percentage of fear in younger female mice during conditioned stimulus recall (acoustic, different context). There were no significant differences between the two groups during the acoustic stimulus trials. However, at 24 h post‐training (8 days after acquisition), WT mice exhibited significantly higher total fear than TgQBP1 mice (*p* = 0.016). (D) Percentage of fear in adult female mice during aversive conditioning. Both groups learned the conditioning task significantly during acquisition (*p* < 0.001), with TgQBP1 females showing significantly lower global fear compared to WT mice (*p* < 0.001). However, at 24 h post‐training, no differences were observed between the groups, whereas at 72 h, TgQBP1 mice exhibited significantly lower freezing than WT mice (*p* = 0.018). (E) Percentage of fear in adult female mice during conditioned stimulus recall (acoustic, different context). No significant differences were observed between the groups during the acoustic stimulus trials. However, at 24 h post‐training (8 days after acquisition), TgQBP1 mice exhibited significantly reduced freezing compared to WT mice (*p* = 0.023). (F) Effect of sex in adult TgQBP1 mice during aversive conditioning. During acquisition, both groups learned the conditioning task significantly across trials, with no differences between them. However, at 24 h post‐training, adult males showed significantly poorer recall of the fear context compared to adult females (*p* = 0.003), which persisted at 8 days (*p* = 0.004). (G) Effect of sex in younger TgQBP1 mice during aversive conditioning. During acquisition, both groups learned the conditioning task significantly across trials, with no differences between them. This lack of difference persisted at 24 h post‐training, indicating no sex differences in younger animals. (H) Effect of age on aversive memory consolidation in male TgQBP1 mice. Both age groups learned the conditioning task significantly during acquisition (*p* < 0.001), with a significant overall age effect (*p* = 0.044). This effect persisted at 24 and 96 h post‐training, with younger TgQBP1 mice exhibiting significantly higher fear than adults (*p* = 0.052 and *p* = 0.033, respectively). (I) Effect of age on aversive memory consolidation in female TgQBP1 mice. Both age groups learned the conditioning task significantly during acquisition (*p* < 0.001). However, younger females exhibited significantly higher fear than adults during acquisition (interaction *p* = 0.024 and age *p* < 0.001), particularly in Trial 2 (*p* = 0.054), Trial 3 (*p* = 0.025), and Post (*p* = 0.007). Post‐training context review showed that adult females exhibited significantly less fear than younger females at 8 days (*p* = 0.028), indicating greater long‐term memory consolidation impairment in younger females. For comparisons between independent groups: *p < 0.05, **p < 0.01, ***p < 0.001, trends 0.05 ≥ # < 0.09; for comparisons between dependent groups: +*p* < 0.05, ++*p* < 0.01, +++*p* < 0.001, trends 0.05 ≥ #′ < 0.09.

Secondly, considering that women are twice as likely to develop PTSD [10, 82, 83], we decided to analyze the consolidation of aversive memory in female mice. These differences may be related to the predominance of the basal amygdala in fear consolidation in females, who tend to exhibit greater fear response to context and generalized fear [84, 85, 86]. For our study, female mice aged P90 were exposed to contextual fear conditioning, with context revision performed at 24–72 h considering that females exhibit a faster loss of specific context memory [86]. Acquisition indicated that both groups properly learned the aversive conditioning (Trials *p* < 0.001) without differences between them (Figure 5B). The total freezing percentage in aversive context recall (24–72 h) showed that younger TgQBP1 females tended to exhibit significantly less freezing at 24 h compared to WT mice (*p* = 0.066), a difference that remained significant over time (72 h *p* = 0.044). Furthermore, analysis of the conditioned stimulus (acoustic cue, Figure 5C) revealed no significant differences between the groups, but the analysis of aversive context recall after the stimulus in younger female mice (8 days post‐acquisition) maintained the significant difference between groups (*p* = 0.016). These results confirmed the effect of QBP1 on memory consolidation in younger female mice. Similarly, we analyzed the stability of aversive memories in adult female mice (Figure 5D). Both groups significantly learned the conditioning during acquisition (Trials *p* < 0.001), but adult TgQBP1 females exhibited a significantly lower overall freezing behavior during acquisition compared to WT mice (Group *p* < 0.001), with a significant difference in Trial 3 (*p* = 0.034). However, the analysis of aversive context recall at 24 h showed no differences between groups, while the adult TgQBP1 females displayed significantly less total fear than WT mice at 72 h (*p* = 0.018; Figure 5D, blue). Lastly, analysis of the conditioned stimulus recall in adult female mice (auditory cue) showed no significant differences between groups (Figure 5E), despite WT mice performeing higher freezing percentages. A significant difference was observed in the context review at 8 days, where adult TgQBP1 females showed significantly lower total freezing percentages compared to WT mice (*p* = 0.023). Together, these data indicate that the impairment in aversive memory consolidation is also present in adult female TgQBP1 mice.

Next, a comparative analysis of all behavioral data was conducted to draw conclusions regarding the effect of age and sex on the deterioration of aversive memory in TgQBP1 mice. First, the effect of gender was analyzed by comparing each age group separately (Figure 5F–G). In adults, both sexes learned the aversive conditioning significantly without differences between them (Trial *p* < 0.001; Figure 5F). However, adult females showed significantly better recall of the aversive context compared to adult males at 24 h (*p* = 0.003) and 8 days (*p* = 0.004). In younger mice, both sexes also learned the aversive conditioning without significant differences between them (Trial *p* < 0.001; Figure 5G), and this lack of difference persisted in the 24‐h context review. Together, these results suggest that although the ability to learn aversive conditioning is similar across genders and ages, adult females exhibit better retention of aversive memory compared to adult males, a difference not observed in younger mice.

Furthermore, the effect of age was analyzed, separately comparing each sex (Figure 5H,I). In male TgQBP1 mice, the acquisition reflected that both age groups significantly learned the conditioning (Trials *p* < 0.001), with an age effect observed (Age *p* = 0.044; Figure 5H). This significant difference persisted in the analysis of aversive context recall at 24 h (*p* = 0.052) and 96 h (*p* = 0.033), where adult mice showed significantly less fear than younger mice. Regarding female TgQBP1 mice (Figure 5I), the fear acquisition showed a significant interaction between variables (*p* = 0.024), indicating both age groups significantly learned the conditioning (Trials *p* < 0.001) and there was an age effect (Age *p* < 0.001). Specifically, younger females exhibited significantly higher freezing percentages than adult females during acquisition [Trial 2 (*p* = 0.054), Trial 3 (*p* = 0.025), and Post (*p* = 0.007)]. However, during aversive contextual recall analysis, no differences between age groups were observed at 24–72 h, but younger females exhibited significantly less fear than adults at 8 d (*p* = 0.028). This differential behavior could be due to the fact that adult females may have been more affected by exposure to the Cue test on the previous day and, therefore, may be over‐reacting to the context recall on day 8. In conclusion, age is suggested to have a significant impact on aversive memory recall in the TgQBP1 mice of both sexes. Specifically, younger mice tend to show greater freezing behavior during acquisition. However, this effect was reversed during long‐term context reviews, where younger males showed higher freezing percentages.

Along these lines, the analysis of WT mice showed a behavior similar to that of TgQBP1 mice in terms of age and sex (Figure S5). Adult female mice showed significantly more freezing in the analysis of aversive context recall at 24 h and 8 d compared to adult males (*p* = 0.045 and *p* = 0.009 respectively, Figure S5A), whereas younger mice did not show sex differences (Figure S5B). Regarding age, younger WT males showed significantly more freezing than adults in the aversive context analysis at 24–96 h (*p* = 0.061 and *p* = 0.021 respectively, Figure S5C), while no significant age‐related differences were observed in WT females except for a significant increase in freezing at 8 d (*p* = 0.004, Figure S5D). Taken together, the behavior of WT mice is similar to that of TgQBP1 mice, demonstrating that age and sex matter when analyzing memory consolidation, but the presence of QBP1 in the transgenic mice did not promote additional differences.

### 2.6 Behavior of TgQBP1 Mice under a PTSD‐like Protocol

After having characterized memory consolidation in the TgQBP1 mice, for the validation of QBP1 as a potential therapeutic agent for PTSD, we have implemented a PTSD‐like protocol based on classical contextual conditioning, which has been extensively validated [87, 88]. Specifically, we have applied the protocol used by Fernández de Sevilla et al. (2022) [89], which is based on previous methods developed in mice [90, 91]. This model uses the application of traumatic electric footshocks (2.5 mA intensity and five consecutive trials), which allow the development of fear and its persistence over time (re‐experiencing and associative fear) accompanied by PTSD symptoms (anhedonia) (Figure 6A).

**FIGURE 6:**
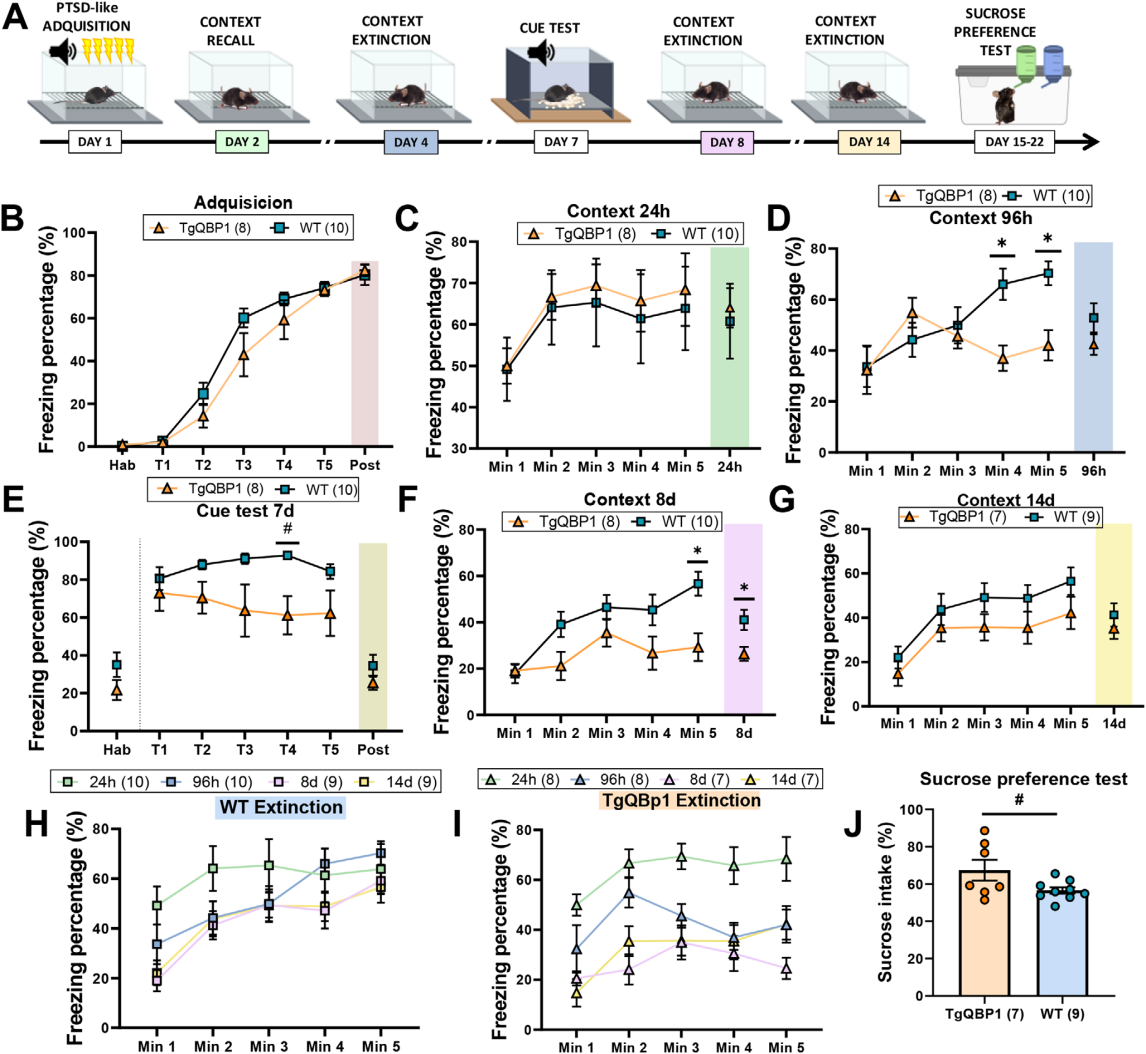
The TgQBP1 mouse extinguishes the conditioning of a PTSD‐like protocol and decreases its freezing. (A) Diagram of the temporal sequence of the PTSD‐like protocol used, based on the contextual fear conditioning test. The acquisition implements five paired foot shocks (2.5 mA, unconditioned stimulus) with a neutral conditioned stimulus (acoustic cue, 80 dB), where the subsequent contextual fear is reviewed on successive days (extinction analysis) and a cue test is performed after a week (associative fear). Finally, the protocol ends by performing a sucrose preference test to measure anhedonia. (B) Freezing percentage in the acquisition of the PTSD‐like protocol, reflecting the increase in percentage across trials of the test for the two groups (Trials *p* < 0.001), with no significant differences between them and reaching very high values of freezing (80%). (C) Freezing percentage in the context review at 24 h after PTSD‐like acquisition. There are no differences between their overall fear behavior and both groups significantly vary their freezing over time (Minutes *p* = 0.003) as their freezing at Min 1 is lower (50%) than in the other minutes (65%). (D) The percentage of freezing in the context review at 96 h after PTSD‐like acquisition showed that there are differences between groups since they change their freezing differently throughout the minutes of testing (Minutes *p* < 0.001) depending on the group to which they belong (Interaction effect *p* < 0.001). This means that only WT mice significantly increase their freezing over minutes [*r* = 0.986, *R* ^2^ = 0.973 *p* = 0.0019], whereas the TgQBP1 mice maintain a more constant overall behavior [*r* = 0.026, *R* ^2^ = 0.0067 *p* = n.s.]. This is reflected in a significant difference between them at Min 4 and Min 5 (*p* = 0.010 and *p* = 0.012 respectively). (E) The Cue test (conditioned stimulus, day 7) after the PTSD‐like protocol showed that neither group modified their behavior across trials (constant freezing), but WT mice significantly exhibited higher discrimination of the conditioned cue (increased global freezing values, Group *p* = 0.031 and Interaction *p* = 0.082). Specifically, there is a trend toward a significant difference between the groups at T4 (*p* = 0.084). There are no differences in the habituation and post‐test phases, where the freezing percentage is reduced to 30%. (F) Freezing percentage in the context review at 8 d after PTSD‐like acquisition showed that there are differences between groups, since they change their freezing throughout the minutes of testing (Minutes *p* < 0.001 and Interaction *p* = 0.005) depending on the group to which they belong (Group *p* = 0.013). Thus, only the WT mice significantly increased their freezing over the minutes [*r* = 0.9093, *R* ^2^ = 0.8267 and *p* = 0.032], whereas the TgQBP1 mice maintained a more constant overall behavior [*r* = 0.3982, *R* ^2^ = 0.1586 and *p* = n.s.]. Specifically, there is a significant difference between the groups in Min 5 (*p* < 0.001) and in the overall freezing average at 8 d (*p* = 0.013). (G) The percentage of freezing in the context review at 14 d after PTSD‐like acquisition showed no differences between groups and a time effect (Minutes *p* < 0.001), since both groups modified their percentage of freezing over time. Certainly, this behavior is paradoxical for TgQBP1 mice, which here showed an incremental behavior and higher values than in the previous days. (H) Intra‐subject analysis of fear extinction per minute for the WT mice. The minute‐by‐minute freezing percentage for each extinction test day (24 h, 96 h, 8 d, and 14 d) reflects that WT mice maintain incremental behavior over time (Minute *p* < 0.001 and Interaction *p* = 0.006) but there is no significant overall extinction of their fear (Extinction *p* = n.s.). Furthermore, there are no globally significant differences between the extinction days analyzed. (I) Intra‐subject analysis of fear extinction per minute for the TgQBP1 mice. The minute‐by‐minute freezing percentage for each extinction test day (24 h, 96 h, 8 d, and 14 d) reflects that TgQBP1 mice significantly extinguish their fear at the global level (Extinction *p* < 0.001) after each day of extinction, with some effect of time (Minutes *p* < 0.001). Specifically, the TgQBP1 mice significantly extinguished their freezing between 24 and 96 h (*p* < 0.001) and between 96 h and 8 d (*p* = 0.001). (J) Percentage of sucrose intake in the sucrose preference test after a PTSD‐like protocol. WT mice were observed to ingest less 2% sucrose than TgQBP1 mice (*p* = 0.056), indicating that they exhibit a higher level of anhedonia. For comparisons between independent groups: *p < 0.05, **p < 0.01, ***p < 0.001, trends 0.05 ≥ # < 0.09; for comparisons between dependent groups: +*p* < 0.05, ++*p* < 0.01, +++*p* < 0.001, trends 0.05 ≥ #′ < 0.09.

First, we have performed the acquisition of the PTSD‐like protocol in P60–P120 male mice groups (Figure 6B), where both of them significantly increased their freezing percentage across trials (Trial *p* < 0.001) with no differences between them. PTSD‐like context revision at 24 h has revealed that both groups recalled the fear context as their freezing percentage increased across test minutes (from Min1 = 50% to Min2‐3‐4‐5 = 65–68%, Minute *p* = 0.003) (Figure 6C), but there are no differences between TgQBP1 and WT mice at their overall behavior (Group *p* = n.s.). On the other hand, the analysis of PTSD‐like context revision at 96 h has revealed that there is an effect of time (Minute *p* < 0.001) and an interaction effect (*p* < 0.001) (Figure 6D), which means that there is a differential effect of time between both groups. The correlation between the freezing behavior and time significantly varies along minutes for WT mice [*r* = 0.986, *R* ^2^ = 0.973 and *p* = 0.0019], while TgQBP1 mice showed a constant behavior [*r* = 0.026, *R* ^2^ = 0.0067 and *p* = n.s.]. Indeed, there is a significant difference between groups at Min 4 (*p* = 0.010) and Min 5 (*p* = 0.012). These first results suggest that there are behavioral differences between the two groups: the WT mice seem to maintain a greater recall of the PTSD‐like context, although the differences appear in the longer term (96 h) compared to a simple aversive memory paradigm (24 h, Figure 4C).

A week after acquisition, the Cue test was performed to analyze the mice conditioned stimulus consolidation (acoustic tone, Figure 6E), where both groups increased their freezing in the presence of the cue (Figure S5E) without differences along trials (Trial *p* = n.s.) but WT mice overall performance was higher than that of the TgQBP1 mice (Group *p* = 0.031). Moreover, there is a trend to show an interaction effect (*p* = 0.082) since each group vary its freezing percentage along trials, especially at the final trials (T4 *p* = 0.084). The following day, a new review of the conditioned context was analyzed (8 d, Figure 6F), where it was shown an interaction effect (*p* = 0.005) that reflects that WT mice overall freezing behavior is higher than that of the TgQBP1 mice (Group *p* = 0.013, specifically at Min 5 *p* < 0.0001). There is also a change on the freezing behavior along time (Minutes *p* < 0.001), but the correlation analysis revealed that only WT mice significantly increased their freezing as they recalled the aversive context [*r* = 0.9093, *R* ^2^ = 0.8267 and *p* = 0.032] while TgQBP1 mice did not [*r* = 0.3982, *R* ^2^ = 0.1586 and *p* = n.s.]. Finally, when we performed a last context revision at day 14 (Figure 6G), we found no differences between groups and there is a time effect (Minutes *p* < 0.0001). In this sense, the correlation showed that WT mice keep changing their freezing along minutes [*r* = 0.8922, *R* ^2^ = 0.7961 and *p* = 0.042] while TgQBP1 mice just tended to do it [*r* = 0.8319, *R* ^2^ = 0.6921 and *p* = 0.080].

Since the absence of extinction is a predominant symptom of PTSD, we also analyzed the extinction behavior under this protocol. According to this, the intra‐group performance across extinction days showed that WT mice did not extinguish its overall behavior (Extinction *p* = n.s. Figure 6H), although there is an interaction effect (*p* = 0.006) that means that its freezing within each extinction day varies along time (Minutes *p* < 0.001). On the other hand, TgQBP1 mice intra‐subject analysis (Figure 6I) revealed that there is a global extinction effect (*p* < 0.001) and its behavior changes across time (Minutes *p* < 0.001). Moreover, the average freezing percentage between extinction days (Figure S5F, orange) showed that TgQBP1 mice extinguished their fear between Post‐24 h (*p* = 0.018), 24–96 h (*p* = 0.004), and a trend on 96 h–8 d (*p* = 0.058) but not between 8 and 14 d (*p* = n.s.) due to their paradoxical increased freezing at day 14 (Figure 6G).

Lastly, we examined the correlation between freezing and time across extinction (Post phase of acquisition to context 14 d, Figure S5G). This correlation shows that both groups significantly reduced their freezing across time, although 70% of this variation of the TgQBP1 group is explained by time effect [*r* = 0.8426, *R* ^2^ = 0.7100 and *p* < 0.001] while just 40% of WT variance is due to time [*r* = 0.6342, *R* ^2^ = 0.4023 and *p* = 0.007]. Taken together, the extinction analysis shows that the WT mouse does not extinguish fear in the PTSD‐like protocol, whereas the TgQBP1 mouse does. Based on the used protocol, it can be suggested that the WT mouse correctly develops the PTSD model, whereas the effect of QBP1 in the TgQBP1 mouse reduces the traumatic impact of this protocol (by reducing the memory consolidation of the event), thus facilitating its extinction.

Finally, a sucrose preference test (Figure 6J and Figure S5H) was also performed 15 days after the PTSD‐like acquisition, so we could measure murine anhedonia as a characteristic symptom of PTSD [92]. The sucrose intake percentage showed that WT mice tended to drink less of the 2% sucrose solution than TgQBP1 mice (*p* = 0.056), indicating that they have developed higher anhedonia. Moreover, WT mice significantly increased the total volume of fluid ingested in comparison with TgQBP1 mice (*p* = 0.011), which also shows greater homogeneity in the behavior of each animal (Coefficient of variation: WT = 12.69% and TgQBP1 = 35.42%). Taken together, these results suggest that the PTSD‐like protocol promotes an absence of extinction and some anhedonia in WT mice, while the presence of QBP1 in the transgenic mice favors the extinction of the fear context, the conditioned cue (acoustic tone) and also tends to reduce the concomitant anhedonia in the TgQBP1 mice.

### 2.7 mCPEB3 Showed Reduced Aggregation in TgQBP1 Mice

Once the effect of QBP1 on mouse memory consolidation was demonstrated, we wanted to study its mechanism of action based on our working hypothesis, i.e., that QBP1 inhibits the amyloidogenesis of CPEB3 and that this blockade is what causes memory impairment. After performing the memory tests, the hippocampi of the mice tested were extracted and mCPEB3 was characterized following a detergent insolubility protocol specific for amyloid proteins [56, 58], assuming that, after undergoing a learning process, mCPEB3 oligomerizes and increases its presence in the insoluble fractions [54, 58]. This protocol uses strong detergents in the lysis buffer and ultracentrifugation to separate the cytosolic material into two fractions (Figure S6A): aggregated protein (amyloid, precipitate P2) or soluble protein (supernatant S2). Western blot analysis of the band corresponding to mCPEB3 (75 kDa) was then performed comparing basal conditions of WT mice (WT naive, Figure 7A) with the conditions after learning in WT and TgQBP1 mice (WT learn and TgQBP1 learn, Figure 7A), comparing the soluble (S2, soluble protein) and precipitated (P2, aggregated protein) fractions corresponding to the cytosol fraction.

**FIGURE 7:**
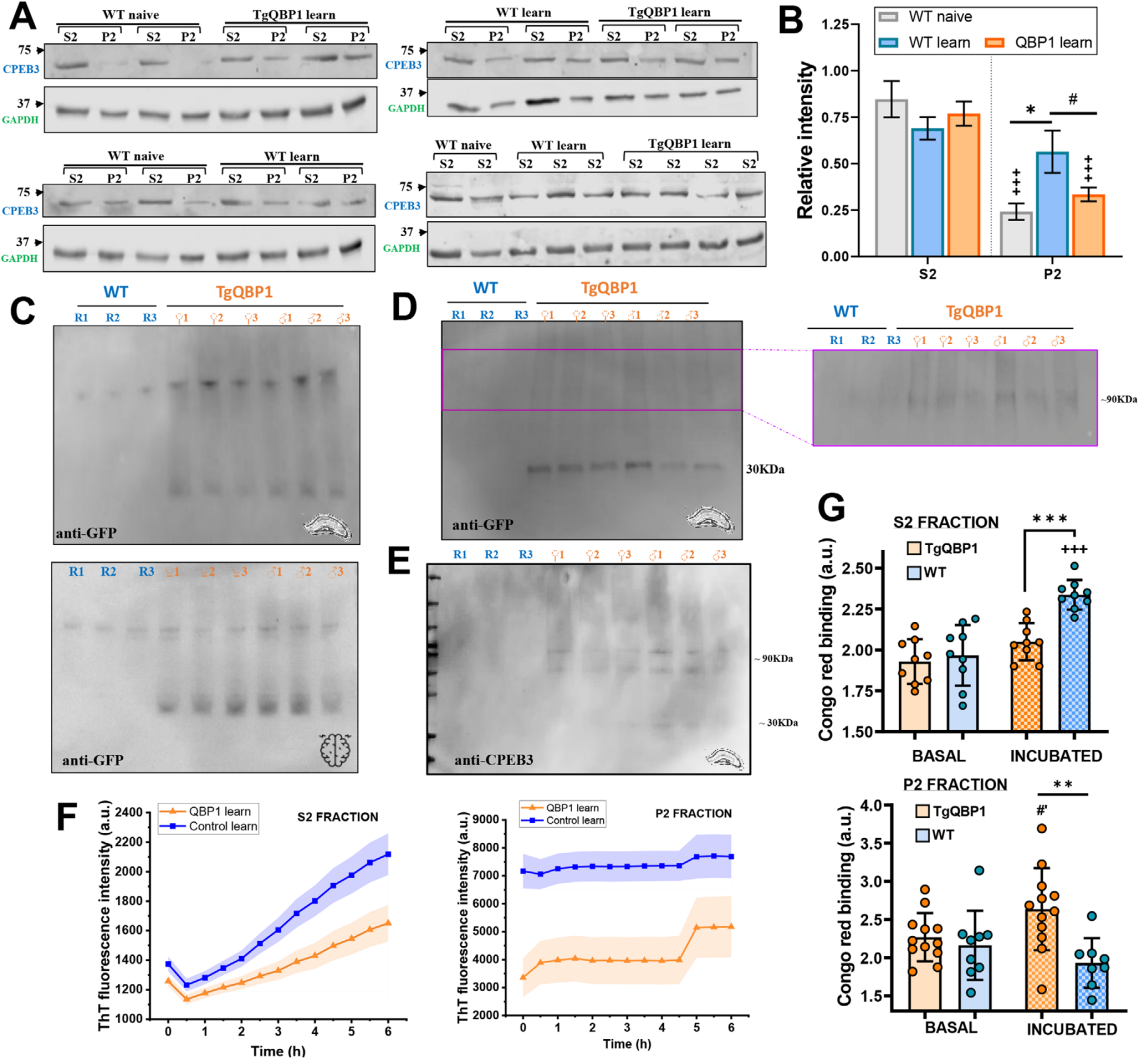
Analysis of mCPEB3 protein in the TgQBP1 mice (learning versus basal conditions). (A) Representative images of Western blots from the analysis of soluble (S2) fractions of hippocampal samples, comparing the learning conditions of both groups for mCPEB3. Recombinant hCPEB3 IDR (1‐450aa) produced in E. coli was used as positive control (50 kDa) and GAPDH as a loading control. (B) Quantification of the 75 kDa band in the S2 and P2 fractions of mCPEB3 in the hippocampus (ImageJ). The statistics show that the soluble fraction (S2) has a significantly higher value than the insoluble fraction (P2) in basal conditions (TgQBP1 *p* = 0.012 and WT *p* = 0.009) and in the TgQBP1 mouse after fear conditioning (*p* = 0.028). Furthermore, the basal TgQBP1 mouse tends to have more soluble protein (S2) than the control (*p* = 0.084); whereas the control mouse tends to have a higher soluble fraction after aversive conditioning compared to basal (*p* = 0.058). Finally, only the control mouse significantly increases its insoluble fraction (amyloid) after aversive conditioning (*p* = 0.021). (C) Native PAGE followed by Western blot against GFP in hippocampal (upper panel) and remaining brain (lower panel) extracts. Two distinct GFP‐reactive bands are observed exclusively in TgQBP1 mouse samples, while a non‐specific high‐molecular‐weight band is seen in WT samples. (D) Co‐immunoprecipitation of hippocampal extracts using GFP‐Trap technology, followed by Western blot detection with anti‐GFP antibody, where a QBP1‐GFPcorresponding band (30 kDa) is detected exclusively in samples from TgQBP1 mice. The right panel shows an extended transfer exposure, revealing a ∼90–100 kDa complex present only in the TgQBP1 samples, consistent with the presence of the QBP1‐GFP complex. (E) Co‐immunoprecipitation of hippocampal extracts using GFP‐Trap technology, followed by Western blot detection with an anti‐CPEB3 antibody; a 50 kDa non‐specific band is observed across all samples. An additional band at ∼90 kDa is predominantly present in samples from TgQBP1 mice, which could be associated with CPEB3/QBP1‐GFP complex formation. (F) Aggregation kinetics of the cytosolic fractions extracted from the hippocampus (S2‐P2) after learning monitored by thioflavin T (ThT) fluorescence intensity. In both cases, the formation of mature amyloid over time is higher for control mice, particularly in the insoluble fraction (P2) where there is a higher proportion of aggregated protein. (G) Amyloid quantification based on Congo Red (CR) binding in the S2 (upper panel) and P2 (lower panel) fractions of hippocampi isolated after learning. CR binding of the soluble fraction (S2) revealed a significant interaction (*p* = 0.024), with significant main effects for both incubation and group (*p* < 0.001 for both). Post‐hoc analysis showed that CR binding in the WT sample significantly increased after incubation (*p* < 0.001) and was significantly higher compared to the TgQBP1 sample (*p* < 0.001). Conversely, the CR binding of the insoluble fraction (P2) shows that only the TgQBP1 sample tended to increase after incubation (*p* = 0.067) and differed significantly from the WT sample (*p* = 0.002). Functional annotation of these proteins revealed a connection to lipid metabolism. Interestingly, Acot1 and Hsd3b4 are also associated with the ovarian steroidogenesis pathway. This finding is particularly noteworthy given that the primary target of this study, CPEB3, is involved in pathways such as oocyte meiosis and progesterone‐mediated oocyte maturation. In contrast, no significant changes were observed in the kidney of TgQBP1 males (Figure S7D). Collectively, these results suggest that the treatment does not induce off‐target proteomic alterations in the hippocampus, with only a limited and specific response in the renal tissue of females involving lipid and steroid‐related pathways.

To analyze possible differences between the groups, quantification of the band corresponding to ∼75 kDa (monomeric canonical form of mCPEB3) was performed in all animal samples analyzed for fractions S2 and P2 (Figure 7B), obtaining the relative intensity value referred to its loading control (GAPDH) for each extraction (Image J software). The analysis reflects that the relative intensity with respect to the mCPEB3 band in the soluble fraction (S2) is similar between groups, with no differences between the WT naive (gray) and both groups after aversive learning (WT learn—blue and TgQBP1 learn—orange, Figure 7B, left). However, the relative intensity in the aggregated fraction (P2) in the WT learn is significantly increased with respect to the WT naive values (*p* = 0.016) and shows a trend in the TgQBP1 learn group (*p* = 0.080) (Figure 7B right). These results show that the insoluble fraction increases when the animal experiences learning, correlated to the aggregation of the mCPEB3 protein needed to perform its function [58]. On the other hand, the presence of QBP1 in the transgenic mice causes an impairment of long‐term memory that is accompanied by a reduction of protein aggregation, similar to the values of WT mice without learning (WT naive). Taken together, these results suggest that the molecular mechanism underlying the long‐term memory impairment found in the TgQBP1 mice could indeed correspond to a decreased mCPEB3 oligomerization produced by the presence of QBP1.

According to these results, we further verified the formation of the QBP1–CPEB3 complex and analyzed the presence of possible off‐target QBP1 interactions in mice hippocampus and remaining brain. We employed three complementary techniques: native polyacrylamide gel electrophoresis (Native PAGE) followed by immunoblotting, co‐immunoprecipitation (Co‐IP) using GFP‐Trap beads, and mass spectrometry of the Co‐IP products. Native PAGE was performed under non‐denaturing conditions, preserving the native conformation of proteins and the integrity of protein complexes. After incubation with an anti‐GFP antibody, a low‐molecular‐weight band was detected exclusively in TgQBP1 mice, both in the hippocampus (Figure 7C, upper panel) and remaining brain (Figure 7C, lower panel), corresponding to the GFP protein. Additionally, a second band appeared at a higher molecular weight, which may represent the QBP1–CPEB3 complex. In control mice, a faint nonspecific signal was detected; however, this band was markedly more intense in TgQBP1 mice. Regarding CPEB3, two bands were again observed. In the hippocampus (Figure S6B, left panel), the lower band was predominant in control mice but nearly absent in TgQBP1 mice, suggesting efficient binding of QBP1 to CPEB3. In contrast, this difference was less evident in the remaining brain (Figure S6B, right panel).

Subsequently, we performed a co‐immunoprecipitation using GFP‐Trap beads, which are specifically optimized for GFP detection. To preserve complex stability, samples were crosslinked prior to the procedure, and kidney tissue was included as a CPEB3 low‐expression control. After incubation with the anti‐GFP antibody, clear bands at approximately 30 kDa were observed in both the hippocampus (Figure 7D) and kidney (Figure S6C, left panel). Upon extended exposure, an additional band at ∼90–100 kDa appeared (Figure 7D, extension), suggesting the presence of a GFP–protein complex, most likely GFP–CPEB3, with no other discernible bands. Moreover, CPEB3 was detected only in the hippocampus (Figure 7E) showing as two higher‐molecular‐weight bands (∼80–90 kDa) and a faint band at ∼30 kDa, further supporting the formation of the GFP–CPEB3 complex. As expected, based on its low expression in kidney, no CPEB3 signal was detected in the kidney (Figure S6C, right panel).

To assess the potential systemic effects of QBP1 overexpression, we performed mass spectrometry of the Co‐IP products and a comparative proteomic analysis of the hypothalamus and kidney. In the hippocampus, no proteins reached statistical significance (FDR < 0.05) in either sex (Figure S7A,B), indicating a lack of detectable off‐target effects. Moreover, within the limits of Co‐IP/MS detection, CPEB3 was identified as a major interacting partner of QBP1 in the hippocampus without widespread detectable off‐target proteomic alterations under these conditions. In the kidney, while the overall proteomic profile remained largely stable, a small subset of proteins showed significant differential expression in TgQBP1 females (Figure S7C). Specifically, Acot1, Hsd3b4, and Gk were found to be significantly upregulated.

### 2.8 QBP1 Reduces mCPEB3 Amyloid State in TgQBP1 Mice After Learning

In order to analyze the amyloid properties of CPEB3 in the mice brain, hippocampal samples isolated and extracted after learning were characterized. Firstly, amyloid propensity was characterized by ThT fluorescence aggregation kinetics, where higher values indicate greater aggregation potential. The results indicate minimal differences in the cytosolic fraction (S1), but in the insoluble fraction (P1), the WT sample aggregated more than that from the TgQBP1 mice (Figure S8A). Furthermore, this difference was maintained in the S2 (soluble protein) and P2 (aggregated protein) fractions from animals that underwent learning (Figure 7F), suggesting that samples from WT mice show greater amyloidogenic potential and aggregation compared to samples where QBP1 is present.

Next, the aggregation potential was evaluated through its binding to Congo Red, a specific staining characterized by a shift in its absorption peak from 490 to 512 nm and an additional peak at 540 nm upon its binding to the mature amyloid structure [93, 94]. In this case, an additional incubation (6 h at 37 °C) was performed to evince the amyloid potential of the sample. After binding quantification (Figure 7G, upper panel), S2 fraction (soluble protein) showed that the sample from WT mice significantly increased its binding after incubation (*p* < 0.001) compared to the TgQBP1 mice sample (*p* < 0.001). The P2 fraction (aggregated protein, Figure 7G, lower panel) exhibited fewer differences between groups, with the TgQBP1 sample significantly tending to increase after incubation (*p* = 0.067) and differing significantly from the WT incubated sample (*p* = 0.002). Taken together, these results suggest that the samples with the highest amyloid potential are the S2 fraction from WT mice and the P2 fraction from TgQBP1 mice, indicating that they contain the highest proportion of protein species able to aggregate. The fact that the P2 fraction (aggregated protein) of TgQBP1 mice maintains an increased amyloid potential suggests that the presence of QBP1 has blocked its amyloid conversion.

Finally, the insoluble fractions (P1–P2) of the learned samples were characterized using immunodot blot against A11/OC conformational antibodies, with CPEB3 as a control (Figure S8B), followed by quantification using Image J (Figure S8C). Quantification did not reveal any significant differences between groups in A11/OC reactivity, although differences were observed across fractions (A11 *p* = 0.005 and OC *p* = 0.002). However, CPEB3 reactivity indicated no interaction effect but showed significant group and fraction effects (*p* < 0.001 both), with TgQBP1 mice displaying a significant increase in P1 reactivity (*p* = 0.007). This difference suggests that mCPEB3 in TgQBP1 samples is less aggregated and more accessible to antibody interaction within insoluble fractions. Taken together, this biochemical and amyloid characterization suggests that the presence of QBP1 in the TgQBP1 mice is decreasing the amyloid potential of CPEB3, evincing that the mechanism of action of QBP1 involves the amyloid blockade of the protein.

## 3 Discussion

Considering the relevance of the amyloid state of CPEB3 in memory consolidation [45, 58, 95] and the tight control that exists over its functional conversion [56, 57, 96], this protein stands as a molecular switch of long‐term memory consolidation. We already proposed the CPEB *Drosophila* ortholog, Orb2, as a therapeutic target to impair memory consolidation [72] and as a potential approach toward PTSD treatment, as this disorder is caused by the poor adaptation of the brain to a traumatic memory [16, 97]. Considering that PTSD's current pharmacology is mostly based on reducing the associated symptomatology [4, 17, 98], the QBP1 peptide [61, 63] was chosen as a potential compound for a pharmacological intervention as it has already been shown to be effective against other amyloid proteins with polyQ regions by blocking the amyloid conversion of their monomer [67, 68, 99].

We have shown that the QBP1‐M8 peptide effectively inhibits the amyloid state of the human CPEB3 protein. Specifically, it inhibits the aggregation kinetics of the IDR region and its amyloid core (residues 101–200) at ratios 1:4 and 1:8, similar to TDP‐43 [68] and Orb2A [72]. Notably, TEM visualization reveals that the interaction of QBP1‐M8 with both CPEB3 and Orb2A [72] leads to an increase in nonspecific aggregates rather than the formation of mature amyloid fibers. However, QBP1‐M8 effectively inhibits the amyloid core fibrillation (residues 101–200), but it does not inhibit the entire amyloid‐forming subdomain (residues 1–200) (Figure 1E). According to this, the QBP1‐M8 effect could depend on the structure adopted by each fragment. On one hand, the amyloid core is composed of a hydrophobic region with rigid β‐sheet‐rich segments [100, 101, 102] and a Pro/Ala/Gln‐rich region similar to the region characterized in TDP‐43 (341–357) [68]. This means that their glutamines would remain accessible for interaction with QBP1 [68, 103]. On the other hand, the amyloid subdomain (R1_1–200_) is composed of two long Pro/Ala/Gln‐rich regions flanking the hydrophobic region, whose organization into different α‐helices would end up promoting its amyloid aggregation [102], leading to the acquisition of a folded structure inaccessible to QBP1‐M8. Related to this, we have shown that the SCR‐C peptide (QBP1‐M8 random sequence) is also capable of inhibiting the amyloid kinetics of hCPEB3 IDR, as occurs in TDP‐43 [68] and contrary to what occurs with Orb2A and *Ap*CPEB [72, 104]. Considering that the IDRs of TDP‐43 and CPEB3 are based on short Q/N‐rich regions, whereas those of *Ap*CPEB and Orb2 are mostly composed by long glutamine tracts [68, 70, 73, 104], we suggest that the polyvalence and affinity of QBP1‐M8 inhibition may lie on its structure (hydrophobic pocket and tryptophan arrangement) [70], while the SCR‐C mechanism may depend on the specific interaction between its tryptophan residues and asparagine and hydrophobic residues present on the Q/N‐rich IDRs. The current literature supports this hypothesis, where the development of novel anti‐amyloidogenic peptides against the low complexity domain of TDP‐43 [105], suggests that the presence of a dense aromatic cluster rich in tryptophan residues (likely shared by QBP1‐M8, SCR‐C and 1b2‐30‐alt6 peptides) could be key in increasing the specific interaction with proteins rich in Q/N regions.

The generation and characterization of a transgenic mouse constitutively expressing QBP1 reported in this study constitutes the first proof‐of‐concept in mammals that this peptide is a good candidate for investigating future therapeutic approaches for PTSD. Phenotypic characterization of TgQBP1 mice has revealed normal function and development, with a correct neurological profile. However, analysis of memory consolidation has evidenced several long‐term impairments, consistent with previous results in *Drosophila* [66, 72, 104]. Specifically, TgQBP1 mice showed different consolidation and recall behaviors depending on the type of learning: at a short and simple learning process, TgQBP1 mice have shown a concrete memory consolidation impairment at 24‐h revision (locomotor activity—Figure 2B, Novel object recognition—Figure 2D, Morris Water Maze—Figures 2H,I and 3A,B, and Contextual Fear conditioning—Figure 4C). However, if the test involves a longer and repetitive learning process, TgQBP1 mice are able to partially consolidate the task, but their recall is reduced or even unavailable: TgQBP1 mice exhibit impaired memory retrieval, evinced by an incremental freezing behavior in the Context test (CFC, Figure 4C and E) and poorer initial trial performance during Morris Water Maze acquisition and IAR analysis (Figure 3). Moreover, the PTSD‐like results (Figure 6) evince that, under this strong and repetitive aversive protocol, TgQBP1 mice are able to partially consolidate the aversive memory, but perform an extinction of the PTSD‐like conditioning as their memory consolidation is reduced, recall is impaired at the long term (96 h–8 d) and this prevents the onset of PTSD. These results highlight that the type and number of exposures of each learning event influence the reinforcement of memory consolidation [106], which, in turn, could be related to the fact that each time synaptic stimulation associated with learning occurs [45, 107, 108], it triggers the machinery for new protein synthesis and the amyloidogenesis of CPEB3 [58, 109, 110]. According to that, repetitive learning could promote the accumulation of the amyloid state of CPEB3, as a result of its self‐sustaining prion‐like properties [54, 101]. This suggests that the effect of QBP1 on the consolidation of repetitive learning may be reduced, as a result of a pre‐existing pool of active amyloid CPEB3.

We have then examined the mechanism of action underlying the blockade of memory consolidation in the TgQBP1 mice by analyzing the mCPEB3 protein as a therapeutic target for the QBP1 peptide. In hippocampal samples, the band corresponding to mCPEB3 (75 kDa) [49, 111, 112] reflects an increase in the fractions corresponding to protein aggregated in the cytosol (P2) only in WT mice after an aversive learning (WT learn). On the contrary, values of TgQBP1 mice after learning remain similar to those of WT mice without learning (WT naive). Subsequent examination of the amyloid potential within samples derived from both groups evidenced that WT mice samples aggregated significantly more than TgQBP1 mice samples, showing the underlying inhibition of the QBP1 peptide on the amyloid state of the protein. Taken together, these results confirm that mCPEB3 increases its amyloid and aggregated state after learning [58], but the presence of QBP1 limits the acquisition of this active amyloid state in the TgQBP1 mice, which in turn impairs memory consolidation. Moreover, our Co‐IP/MS data highlight CPEB3 as a highly prominent target for QBP1 in the hippocampus without causing widespread proteomic alterations. Nevertheless, although these conditions argue against major off‐target effects, the technical limits of detection mean that minor or transient interactions cannot be fully excluded.

Moreover, our results showed that QBP1 is capable of reducing memory consolidation independently of sex and age, since TgQBP1 mice showed a long‐term memory impairment in each analyzed condition. However, it is worth mentioning certain differences in behavioral responses regarding sex and age, although they are also present in WT mice as well. Our results have shown that both WT and TgQBP1 adult females maintain freezing levels significantly higher than those of males in the context test, while no significant differences are observed in younger animals. Current studies have described similar sex differences for fear responses [85, 113], mostly related to the increased amygdala activation that underlies fear responses in females as opposed to the shift toward hippocampal activation made by males during the expression of contextual fear [82, 114]. In terms of age, impaired memory consolidation appears significantly more pronounced in younger animals compared to adults, potentially due to an age‐related compensatory mechanism. This compensation could be explained by the cumulative activation of cellular machinery each time a memory consolidation attempt occurs. Such repeated activation may promote transient engagement of synaptic plasticity pathways [107, 115], although these mechanisms typically do not persist over time in the absence of consolidation‐associated stimuli (e.g., “labels” or synaptic tags) [107, 108]. Over time, processes like synaptic plasticity and the formation of new synaptic contacts tend to diminish, particularly as learning extinction progresses [116]. However, events independent of de novo protein synthesis, such as intracellular signaling or cytoskeletal remodeling, may persist [117]. These lasting molecular changes are the ones that could facilitate a form of sensitization or compensatory plasticity in older animals, ultimately mitigating the impact of memory consolidation impairments in comparison to younger animals.

As an important derivative of these results, the TgQBP1 mouse represents as an interesting opportunity to clarify the current controversy on the role of CPEB3: for some authors, CPEB3 is considered a repressor of protein synthesis and its blockade would enhance memory consolidation [48, 95, 118], while for others it is an activator of protein synthesis and its blockade would impair memory consolidation [56, 57, 58]. Figure 8 lists all existing mice models described in the literature related to CPEB3 modulation. When CPEB3 expression is abolished (KO CPEB3) [95], the effect on the phenotype relies on the absence of its repressor state, thus resulting in improved learning and an enhanced long‐term memory. Conversely, if CPEB3 expression is specifically abolished after learning (cKO CPEB3) [58], the phenotype relies in the absence of its active state, which results in an impairment in 24‐h memory retention and a spatial cognitive deficit in long‐term platform localization. Finally, when the positive post‐translational modification (Neurl1DN mouse model) [57] or the amyloidogenic state of CPEB3 (TgQBP1) is modulated, the active state of CPEB3 is blocked and promotes impaired long‐term memory and reduced cognitive flexibility and spatial discrimination. Taken together, we suggest that the properties of its active state are the ones that determine its effect on memory consolidation [96, 109], rather than the presence of the protein itself. Therefore, we conclude that CPEB3 appears to be a molecular switch for memory consolidation that exerts a dual function depending on its conformational state, where both states are equally relevant for memory consolidation and synaptic plasticity associated with learning.

**FIGURE 8:**
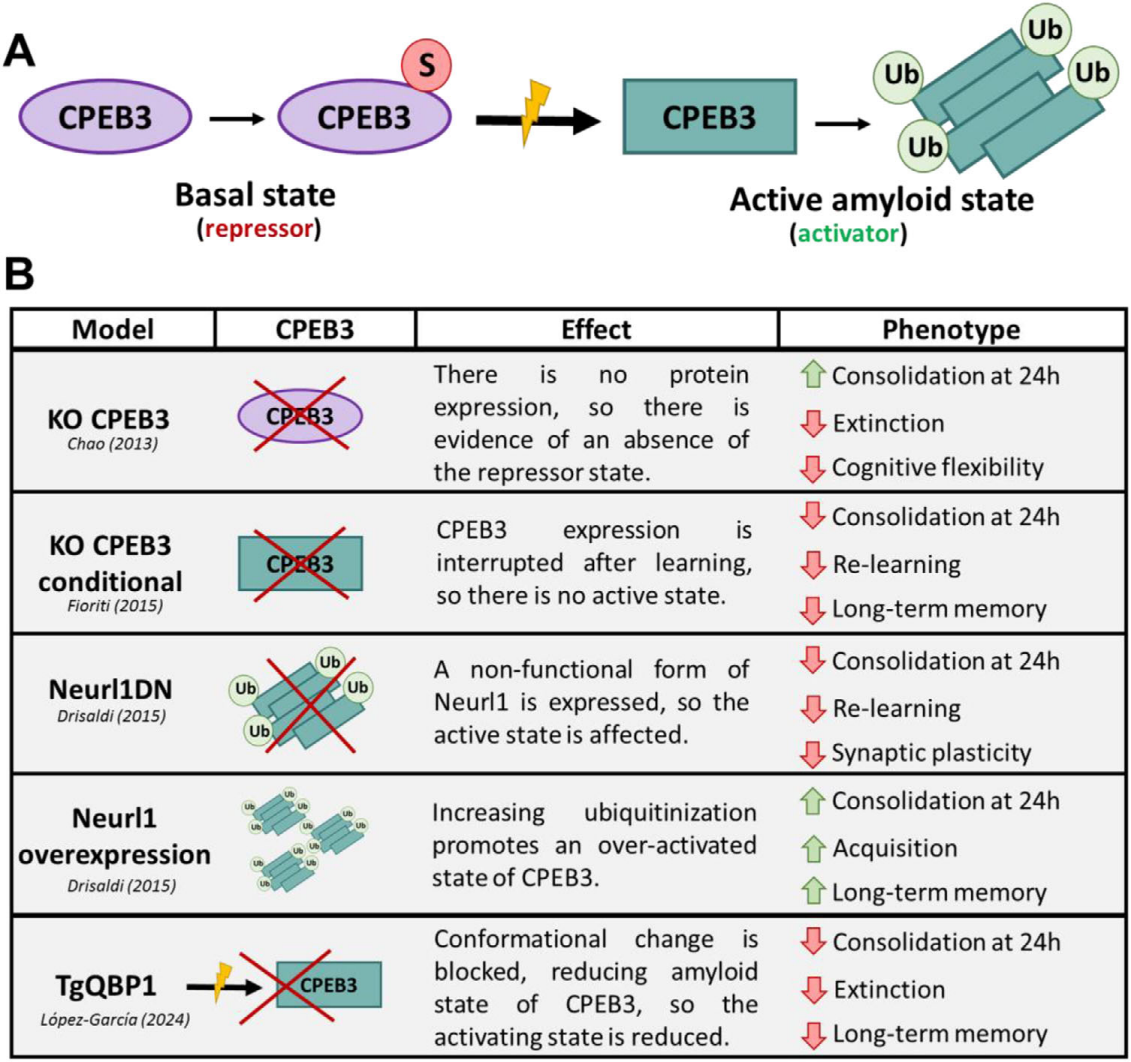
Comparison of the state of the CPEB3 protein modulation in mouse models. (A) Schematics of the CPEB3 conformational change, illustrating the amyloidogenic modulation of the protein depending on its state. CPEB3 is mostly found on its repressive monomeric state in basal conditions, with a sumoylation as a constraint that, after synaptic stimulation, gives raise to the prion‐like/amyloidogenic state that is ubiquitinated (positive posttranslational modification) and acts as an activator of synaptic plasticity by promoting mRNAs translation. (B) Table summarizing the CPEB3 mouse models found in the literature, indicating the modification involved on the CPEB3 protein and the repressor/activator function that is altered, which is directly reflected in a mouse memory phenotype.

Regarding PTSD, the literature has recently established *Cpeb3* as a potential risk gene for PTSD after evaluating the exacerbated and non‐extinguishable fear response in the CPEB3 KO mice [48]. Since this is a model that completely lacks CPEB3 protein [95, 118] and only a phenotype where there is no basal CPEB3 repressor state is present (Figure 8B), the increased susceptibility to fear may actually be due to the potentiation of memory acquisition that arises in this model and which occurs also for tasks where no aversive memories are involved. Our results showed that the presence of QBP1 in the transgenic mice not only impairs hippocampal‐dependent and simple aversive memory but also prevents the onset of PTSD‐like symptoms (Figure 6) by reducing the traumatic impact of the conditioning (impaired memory consolidation and recall) and developing less anhedonia compared to control. Altogether, our results suggest that QBP1 peptide not only decreases memory consolidation (by blocking CPEB3) but also reduces the negative emotional impact of the traumatic event. Ultimately, both studies demonstrate that CPEB3 is involved in memory consolidation and that, depending on how learning occurs and under what conditions [48, 58, 95, 106], these memories will have a greater or lesser emotional impact [16, 31, 119].

Regarding the future preclinical development of QBP1 for PTSD, our results from TgQBP1 mice under the PTSD‐like protocol (Figure 6) suggest that the presence of QBP1 significantly influences the memory consolidation and the occurrence of PTSD‐like behaviors: thus, TgQBP1 mice learned the fear conditioning but their memory impairment favors the extinction of the traumatic event after 96 h (mostly due to a stronger and repetitive learning), which is maintained in the long term and is accompanied by less anhedonia. This suggests that the presence of QBP1 is reducing the aversive impact and allows a response that would be usual for a simple aversive memory consolidation but does not develop PTSD‐like symptoms as the WT mouse does. Therefore, we suggest that QBP1 can be considered as a potential compound to develop a causal treatment for the disorder. While our constitutive model demonstrates the efficacy of chronic QBP1 expression, future studies utilizing inducible post‐developmental expression or direct pharmacological administration will be essential to translate these findings into a practical therapeutic intervention, thereby addressing the current need for an effective pharmacological treatment for PTSD [23, 44, 120]. If we look at the current pharmacology based on fear memory [16, 26, 119], propranolol might be the most comparable drug to QBP1, since it affects the reconsolidation process by indirectly blocking protein synthesis [26, 42]. In mice, the effects of propranolol on both contextual and fear memories have been demonstrated [42, 121, 122], but unfortunately it has failed to prevent PTSD‐like features in rats [42, 123] and has shown mixed results in human trials [4, 6, 41, 124]. On the other hand, hydrocortisone has shown beneficial effects in both rodents and humans [4, 36, 37, 40], which makes it the most promising fear‐based drug for the treatment of PTSD today. Considering that the behavioral protocols used in mice to evaluate the effects of propranolol and hydrocortisone [42, 123, 125] are similar to those we have used here for TgQBP1 mice at aversive memory (Figure 4), which are even more pronounced and specific according to the results of the PTSD‐like protocol (Figure 6). Thus, QBP1 constitutes a promising alternative showing an effect comparable to that of propranolol and hydrocortisone, with the advantage that it affects a direct target that interferes with protein synthesis (CPEB3) to disrupt memory consolidation instead of just modulating the emotional response associated with such memory (i.e., stress response axis disruption).

## 4 Conclusions

At the level of basic research, our study has directly demonstrated the amyloidogenic hypothesis for neuronal CPEB3 in a mammalian system; meanwhile, in terms of biomedical research, we have provided the biological proof of concept that the anti‐amyloidogenic QBP1 peptide can block memory consolidation in mice, thereby demonstrating its effectiveness in mammals. Moreover, our results provide a very encouraging starting point since TgQBP1 mice showed resistance to developing PTSD‐like symptoms, evidencing both extinction of the traumatic event and a reduction in associated anhedonia. Thus, QBP1 constitutes a promising new pharmacological tool that is very promising for the development of new therapies based on the amyloid inhibition of CPEB3, without detectable widespread off‐target interactions under the conditions tested. In addition to demonstrating the safety of the peptide through its constitutive expression, we have shown how QBP1 affects the consolidation of new contextual and aversive memories and reduces the onset of PTSD‐like behaviors, which points to QBP1 as a strong future potential alternative to hydrocortisone and propranolol in fear‐based approaches for PTSD, provided its translational application is validated via acute pharmacological delivery.

## 5 Materials and Methods

### 5.1 Production of the QBP1 Construct

The insert expressing two copies of QBP1 (in tandem) fused to eGFP was cloned into the pCAGGS expression vector, which contains the CAG promoter and the rabbit *β*‐globin splicing intron, terminating with polyadenylation sites. The cDNA encoding QBP1(x2) was obtained from a plasmid isolated by phage display and used for protein expression in cell culture [61], which was fused to the cDNA encoding EGFP. The sequence of all constructs was confirmed by DNA sequencing.

Subsequently, the QBP1(x2)‐EGFP cDNA was inserted in the p‐EGFP‐N1 vector as a template for its production, and then it was amplified to introduce the cDNA insert into the pCAGGS vector. The entire insert with the promoter and the coding sequence of interest was excised with SalI and PstI from the expression vector. This was performed following protocols similar to those previously described for other QBP1 constructs [61, 64].

### 5.2 Production of QBP1 Transgenic Mouse

Fertilized eggs derived from a C57 × C57 cross were extracted from the oviduct and microinjected with the purified cDNA of the insert (enhancer‐promoter‐QBP1 × 2‐EGFP‐polyA terminator), by a subcontracted company (Japan SLC, Inc.). A total of 200 injected ovules were implanted in pseudopregnant females which resulted in the birth of 3 lines of transgenic newborn mice. The incorporation of the transgene was examined by Western blot in the brain lysatesere: brains were homogenized in 500 µL of TBS (pH 6.8) with a protease inhibitor cocktail (Nacalai Tesque). About 10 strokes using a 2 mL homogenizer were sufficient. The lysate was placed in a 2 mL tube and 600 µL of 2 × SDS sample buffer were added using a cut tip. The lysate was pipetted about 5 times, boiled for 10 min, and cooled at room temperature. A 5‐µL aliquot of the lysate was mixed with 20 µL of SDS sample buffer, and then 10 µL were used for SDS PAGE/Western blotting.

### 5.3 Recombinant Protein Production and Amyloid Characterization

The production of hCPEB3 IDR as a recombinant protein was carried out following the methodology described elsewhere [73]. For amyloid inhibition, QBP1‐M8 (octapeptide: WKWWWPGIF) and SCR‐C (octapeptide: WPIWKGWF) [62, 69, 70] were prepared at 10 mM (stock concentration) in dimethyl sulfoxide (DMSO, Sigma‐Aldrich 276855) to facilitate their dissolution. Visualization of amyloid fibers was performed by transfer electron microscopy (TEM), where 5 µL of incubated protein (different times and concentrations) were adsorbed on carbon‐coated 300‐mesh copper grids (Ted Pella Inc.) and subsequently were stained with 10 µL of 1% (w/v) aqueous uranyl acetate solution. The analysis was performed at 80 kV excitation voltage using a JEOL JEM 1200 EX II TEM equipped with a Megaview III CCD camera. Amyloid aggregation was monitored by performing ThT binding kinetics, analyzing ThT fluorescence intensity changes after binding to mature fibers [74]. For this purpose, the reaction was prepared by mixing the sample at the desired concentration with 50 µM ThT (the stock was prepared at 1 mM concentration in PBS buffer), recording fluorescence intensity measurements over time on a FLUOstar OPTIMA plate reader at 37 °C with excitation at 440 nm and emission at 490 nm. The experimental values of ThT fluorescence intensity of the samples were subtracted from the value obtained for the blank (PBS‐ThT buffer). Specific details of the analysis of the homogenized fraction of seahorses are provided in the *Supporting Information* section.

### 5.4 Animals

The experiments were carried out in male and female QBP1 transgenic mice of constitutive expression from postnatal day 90 (P90, younger mice) to 4–5 months (adult) of age and wild type littermates C57BL/6Ola (WT). The mice were generated from frozen embryos donated by Prof. Yoshitaka Nagai (previously frozen by the in‐house facility of National Center of Neurology and Psychiatry), starting their analysis from the third homozygous generation, whose expression of the transgene was already stable. Frozen embryos were recovered by the Mouse Embryo Cryopreservation Facility at the Centro Nacional de Biotecnología‐CSIC (Madrid). The mice were housed in groups of 4–6 per cage, under conditions of constant temperature (21–22 °C) and a light/dark cycle of 12 h; with food and water ad libitum. The animals were treated following the guidelines contained in RD 53/2013 and in accordance with European Community Guidelines (Directive 2010/63/EU), previously approved by the Bioethics Committee of the Cajal Institute, the CSIC and the Community of Madrid.

Genotyping was carried out by the Molecular and Cellular Biology Unit of the Cajal Institute, using mouse tail tissue (Illustra tissue and cells Genomic prep kit, GE Healthcare) and the copy number was quantified by quantitative PCR (qPCR, Taqman Copy Number assays, Applied Biosystems). A general protocol was followed with a basic battery of behavioral and neurological characterization, as described elsewhere [126], provided by the Unit of Animal Behaviour at the Cajal Institute. Weight, temperature and abnormal physical characteristics were weekly measured (bald spots, excessive grooming and integrity of the vibrissae).

### 5.5 Novel Object Recognition Test (NOR)

NOR is a test that allows analyzing the discrimination of new objects with respect to objects already known, in a protocol of three phases: training, short‐term test (both in the first day) and long‐term test (second day) (*N*_QBP1_ = 9 and *N*_WT_ = 10). The protocol and the guidelines used were as described elsewhere [127]. Mice were first habituated in the arena for 15 min and exposed to two different objects (A–B, training); 1 h later short‐term memory was tested for 10 min, in which mice were exposed to A–C objects. 24 h later, long‐term memory was tested (10 min) and mice were exposed to A–D objects. The mice were recorded using a video camera and the exploration time was counted using EthoVision XT 8.5 software: the observer who blindly analyzed the results considered the active approach of the mouse nose within a one centimeter range of the object as an exploration of the object. The discrimination index (DI) was determined by the difference in exploration time expressed as a ratio of the total time spent exploring the two objects: good discrimination was settled at 0.20 DI value, considering that there must be significant differences between the training and the testing phases of the test.

### 5.6 Morris Water Maze (MWM)

After a habituation test (day 0) in which preferences between quadrants in the different experimental groups were ruled out, the animals learned to find a hidden platform during the following 5 days, through 4 trials/day (60 s each trial, plus 20 s on the platform). If an animal could not reach the platform, the experimenter placed it on top of it. Subsequently, the animals were subjected to a trial on the seventh day, without the platform, to assess preference for the platform quadrant. During this test the trajectories were analyzed to measure time spent swimming in the platform quadrant (Probe Q) compared to time in the other three quadrants, or time spent swimming above the virtual platform position (Probe P) compared to time above the virtual mirror or platform positions in the other three quadrants. On days 17 and 18, animals were exposed to a new series of four trials daily for two days, recording escape latency. To analyze the internal performance within each experimental day, we have developed a new variable called Inter‐trial Acquisition Ratio (IAR, Figure 3C) to quantitatively compare the performance of the mice in Trial 1 with respect to the average of the four trials of each acquisition day.

### 5.7 Contextual Fear Conditioning (CFC)

To analyze aversive memories, fear conditioning was established in the animals using the UGO BASILE ANY‐maze controlled fear conditioning system (mouse cage). On the acquisition day, after the adaptation time (180 s) the mice were exposed to three 30 s trials: a light electric shock on the paw was used as the unconditioned stimulus (UCS, 0.8 mA intensity) and administered in the last 2 s of the trial, in which a conditioned stimulus (CS, 30 s continuous white noise −70 dB) was present. Each trial was spaced by a fixed inter‐trial interval (50 s) and then recorded for a final 30 s after the last trial (Post). 24 h after acquisition, the context test was evaluated: mice were returned to the conditioning chamber and freezing behavior was measured for 5 min (no shock and 250 lux). Likewise, 7 days after acquisition, the recall of the conditioned stimulus (acoustic cue) was evaluated, using the same protocol as during fear implantation, but eliminating the unconditioned stimulus (electric shock). Extinction of fear to the conditioned context was assessed at 96 h and at 8 days after fear acquisition using the same protocol as the context test. The experimental design and subsequent data collection were performed using the behavioral tracking program associated with the equipment (ANY‐maze software).

To implement the PTSD‐like protocol based on fear conditioning, we have adapted protocols previously validated in mice [89, 90]. On the acquisition day, after the habituation time (120 s), mice were exposed to 5 consecutive trials of 30 s, where a neutral conditioned stimulus (CS, 30 s continuous acoustic cue −80 dB) was paired with a severe electric foot shock (UCS, 2.5 mA intensity) in the last 2 s of the trial. Each trial was followed by longer inter‐trial interval (90 s) and then mice were left in the chamber for a further 5 min (to allow them to calm down). As mentioned above, contextual conditioned recall was assessed on subsequent days (24 h, 4 d, 8 d, and 14 d) to evaluate extinction and recall of the conditioned stimulus (acoustic cue) a week after acquisition. Finally, on day 15 of the protocol, a sucrose preference test [128] is performed to measure anhedonia in mice (a hallmark PTSD‐related symptom).

### 5.8 Statistical Analysis

Statistical analysis was performed with IBM SPSS Statistics (Version 26.0, Armonk, NY, IBM Corp) and GraphPad Prism (version 8.0.2). First, the normal distribution of the data was analyzed (Shapiro‐Wilk, *N* < 50), along with the homogeneity of their variances and the identification of extreme values, which were removed only in case they were considered extreme cases by the analytical software. Subsequently, the values were analyzed according to the number of variables and groups to be compared. In the case of comparing two samples. Student's *t*‐test (unpaired), or the Mann–Whitney *U*‐test (nonparametric cases) was used in case of independent samples; whereas Student's *t*‐test (paired) or the Wilcoxon signed‐rank test (nonparametric cases) was used in case of repeated or related measures. In case of more than two samples, a one‐way ANOVA analysis of variance or the Kruskal–Wallis test (nonparametric cases) was used for independent samples, whereas a two‐way ANOVA or mixed model (Bonferroni for posthoc) or Friedman's test was used for related or paired samples. Statistical significance was set at *α* = 0.05. The data were managed in Excel and, subsequently, graphs and images were generated using Origin 2021 and GraphPad Prism 8. For independent group comparisons: *p < 0.05, **p < 0.01, ***p < 0.001, trends 0.05 ≥ # < 0.09; for dependent group comparisons: +*p* < 0.05, ++*p* < 0.01, +++*p* < 0.001, trends 0.05 ≥ #′ < 0.09.

### 5.9 Hippocampi Homogenization and Fractionation

In order to analyze the WT and TgQBP1 mice that performed the hippocampal‐dependent memory tasks, their hippocampi were homogenized using a detergent‐rich protocol [54, 56]. The extracted hippocampi were incubated in 300 µL of lysis buffer (50 mM Tris pH 7.5, 1 mM EDTA, 10 mM KCl, 0.5% Triton X‐100, 0.5% NP40) for 1 h in a cold room. Upon removing a small volume of homogenate for further analysis, the rest of the volume was centrifuged for 5 min at 4000 rpm (Microfugue 18 centrifuge, Beckman Coulter) to obtain the cell debris fraction (P1) and a soluble fraction (S1), from which an aliquot was reserved as well. This supernatant was ultracentrifugated at 65,000 rpm for 1 h at 4 °C in a TLA 100.1 rotor (Beckman Coulter) to obtain the soluble (S2) and insoluble (P2) protein fractions. Both pellet fractions were resuspended in 50 µL of detergent buffer.

### 5.10 Western Blot of Hippocampal Samples

Dissected hippocampi were homogenized in the detergent buffer and fractionated into soluble and insoluble fractions as mentioned. From each fraction, 150 µg of protein were subjected to SDS‐PAGE using 12.5% acrylamide gels and then transferred to a nitrocellulose membrane (0.45 µm, Amersham Protran) in Tris‐glycine buffer with 15% methanol. PBS 0.1% Tween‐20 (PBS‐T) with 5% nonfat milk (Biorad) was used to block the membrane, which was later incubated with the primary antibody: anti‐CPEB3 rabbit polyclonal (ab10883) or anti‐GAPDH rabbit polyclonal (ab37168). Washing steps were carried out with PBS‐T 10% nonfat milk. IRDye 680LT Goat anti‐rabbit (LI‐COR) was used as the secondary antibody and then developed in an Odyssey CLx (LI‐COR) apparatus. The images obtained were analyzed using Image J software to quantify the intensity of the bands corresponding to the mCPEB3 band and the GAPDH loading control, for which several different Western blots were analyzed for each sample (*N* = 5 animals/group). The analysis of these data was performed by calculating the relative intensity comparing the quantification of the mCPEB3 band with that of the GAPDH band (CPEB3/GAPDH) for each transfer and well analyzed, normalizing the quantified intensity for each band to make them comparable.

### 5.11 Coimmunoprecipitation (CoIP)

Tissues were lysed using a specific IP Cell Lysis Buffer (Thermo Fisher, Waltham, MA, USA; Cat. #87787) supplemented with 1 mM PMSF. After 30 min of incubation on ice, the lysates were centrifuged at 12,000 × *g* for 2 min at 4 °C. Protein concentration was determined using the Lowry Protein Assay (BioRad, Hercules, CA, USA; Cat. #5000112). Mouse anti‐GFP antibody was added according to the ratios recommended in the manufacturer's datasheet. The protein–antibody mixture was incubated overnight at 4 °C under gentle rotation. For protein capture, ChromoTek GFP‐Trap (Thermo Fisher, Waltham, MA, USA; Cat. #17313363) was used. The beads were washed twice with PBS containing 0.005% Tween20 (PBSTween) and then incubated with the protein–antibody mixture for 1 h at 4 °C under rotation. Beads were subsequently washed three times with PBSTween and once with deionized water.

For elution, 100 µL of glycine elution buffer (0.1 M glycine, pH 2.6) were added. Samples were agitated at 200 rpm for 10 min at room temperature (RT), magnetized, and the supernatant was collected. The eluate was neutralized with 5 µL of TrisHCl (pH 9.5). A second elution was performed to improve protein recovery, followed by magnetization and transfer of the supernatant to a new microcentrifuge tube for further analysis.

Protein crosslinking in the CoIP: Co‐immunoprecipitated samples used for SDS‐PAGE were crosslinked with DMTMM to preserve complex structure. DMTMM was added to the samples and incubated under gentle agitation for 30 min, resulting in a final concentration of 1 mg/mL. The reaction was then quenched with 10% (v/v) 1 M ammonium bicarbonate and incubated for an additional 30 min.

Extended information on additional experiments and detailed description of the experiments with mice have been included in the *Supporting Information* section.

## Author Contributions

M.C.‐V. conceived the project. P.L.‐G., J.L.T., K.R.M.G., and D.R.M. designed the animal experiments. P.L.‐G., J.L.T., and K.R.M.G. obtained the experimental data, performed its analysis and interpreted the results with the help of A.S. and A.P. Moreover, H.A.P. and Y.N. designed the QBP1 construct and produced the QBP1 transgenic line, and performed the first characterization. D.R.M. recovered and maintained the initial transgenic line, confirming the expression of the peptide in the TgQBP1 mouse hippocampus. P.L.‐G. and M.E.V. designed the biochemical experiments, analyzed the data and interpreted the results. J.M.S.‐R. and M.D.C. performed, analyzed, and interpreted the co‐immunoprecipitation results. Finally, P.L.‐G. wrote the initial draft of the manuscript, all authors reviewed and provided feedback on the manuscript, and M.C.‐V. wrote the final version.

## Conflicts of Interest

MC.‐V. is co‐inventor of a patent regarding QBP1 as a lead compound for PTSD and ASD (PCT/EP2016/057801) and has received funding from the Spanish Ministry of Economy (SAF2013‐49179‐C2‐1‐R and SAF2016‐76678‐C2‐1‐R) and also from the Community of Madrid for the industrial PhD grant awarded to P. López‐García (IND2020/BMD‐17444). The remaining authors declare no competing financial interests or potential conflicts of interest.

## Supporting information




**Supporting File**: advs77862‐sup‐0001‐SuppMat.docx.

## Data Availability

The mass spectrometry proteomics data generated in this study have been deposited to the ProteomeXchange Consortium via the PRIDE partner repository with the dataset identifier PXD078931 (https://doi.org/10.6019/PXD078931).
