# Supplementary material for: An Anti‐Amyloidogenic Approach to Specifically Block Memory Consolidation in Mice for Therapeutic Intervention
Source: Adv Sci (Weinh). 2026 Oct 16:e02541. Online ahead of print. doi: 10.1002/advs.202502541 (PMC13673324; doi:10.1002/advs.202502541)
Supplement: Supplementary file 1 [file ADVS-9999-e02541-s001.docx]

**Supplementary Information**

**An anti-amyloidogenic approach to specifically block memory consolidation in mice for therapeutic intervention**

Paula López-García, Kerry R. McGreevy, Maria Eugenia Vaquero, Juan M. Suarez-Rivero, Antonio Moreno-Rodríguez, Antonio J. Pérez Pulido, Daniel Ramírez de Mingo, Anna Pallé, Helena Akiko Popiel, Andrea Santi, Mario D. Cordero, José Luis Trejo, Yoshitaka Nagai, and Mariano Carrión-Vázquez

***Supplementary experimental data***

**Figure S1. QBP1-M8 induces non-specific aggregation during hCPEB3 fibrillation.**

**A)** Representative TEM micrographs of the amyloid cascade of hCPEB3 R1_1-200_ (10 μM) in the presence or absence of QBP1-M8 (1:10). Samples incubated at 37 ᵒC in PBS pH 7.4 without shaking. The scale bar is indicated on each micrograph.

**B)** Representative images of fibrillation of de hCPEB3 R2_1-300_ (10 μM) in the presence or absence of QBP1-M8 (1:10). Samples incubated at 37 ᵒC in PBS pH 7.4 without shaking. Scale bar is indicated on each micrograph.

**Figure S2: QBP1 transgenic mouse: generation, characterization and QBP1 expression.**

**A)** Schematic representation of the construct used to generate the QBP1 transgenic mouse. This construct was used to microinject fertile eggs into pseudo-pregnant females, using the CAG promoter for its constitutive expression and long-term maintenance of protein expression.

**B)** Western blot of the generated transgenic lines. Of the three lines produced, only one of them (named QBP1-3) confirmed the expression of the peptide in the supernatant and was the one used to generate the breeding mouse. Q19-YFP expressing COS-7 lysate was used as a positive control, revealed with an anti-GFP (1:500) and a secondary mouse IgG-HRP antibody (1:2000).

**C)** Confocal microscopy images of hippocampal dentate gyrus sections obtained from WT and TgQBP1 mice (10 weeks old), in which EGFP was observed for its native fluorescence and Hoechst, as a DNA stain, was used to label cell nuclei. Scale bar: 50 μm.

**D)** General health characterization of the QBP1 transgenic mouse according to Crawley, 1999^1^.

**Figure S3. Extended characterization of long-term memory in the TgQBP1 mouse.**

**A)** Total vertical activity on the two days of the protocol in the activity cage, where on day 1 there was no difference between groups. On re-exposure on day 2, the TgQBP1 mouse significantly increased its activity compared to the WT mouse (p=0.027), despite both significantly decreasing their activity compared to the previous day (WT p<0.001 and TgQBP1 p<0.001).

**B)** Time spent in the open and closed arms of the elevated cross maze in basal conditions, where TgQBP1 mice show normal anxiety behavior. There is no significant difference between groups in total time, where both spent significantly more time in the closed arms than in the open arms (p<0.001).

**C)** Exploration in the elevated cross maze in basal conditions, where the mobility of the animals is evident and they enter significantly more times into the closed arms (TgQBP1 p<0.001 and WT p=0.0012).

**D)** Object Recognition Protocol overview: The test involves a training phase (15 min) where a mouse freely explores a box with two distinct objects. After one hour (short term), the mouse explores the box again (10 min) with one familiar (blue) and one new (yellow) object. Twenty-four hours later (long term), the mouse is reintroduced into the box with the same familiar object (blue) and a new one (pink). The mouse must discriminate the novel object by increased exploration.

**E)** The percentage of preference for novelty reflects that the mice modify their preference as a function of time and group (Interaction p<0.001). The WT mouse increased its preference from training to long term (p=0.002), while the TgQBP1 mouse significantly increases its preference from training to short term (p=0.004) and this significantly decreases from short to long term (p=0.041). This is because in the long term, the WT mouse prefers the novelty significantly more than the TgQBP1 mouse (p=0.003), because it divides its preference between both objects (50-50%). The dash purple line indicates the neutral value of preference (50%), from which novelty is discriminated.

**F)** Performance plot of TgQBP1 mice in test acquisition: the escape latency corresponding to the first trial of each day (Test 1) and the last trial of each day (Test 4) are shown. These data show that the TgQBP1 mouse performs better on each test even within the same day, as evidenced by a significant trend on Day 5 (p=0.063) and a significant difference on Day 17 (p=0.040). Data were analyzed with the Mann Whitney test.

**G)** Performance plot of WT mice in test acquisition: the escape latency corresponding to the first trial of each day (Test 1) with respect to the last test of each day (Test 4) is shown. Data show that the WT mouse maintains a similar performance in all the tests of each day, with no significant differences between them.

**Figure S4: Contextual fear conditioning confirmed the blockade of aversive memories in TgQBP1 mice.**

**A)** Comparison of the average freezing percentage at trials (acoustic cue presence) or ITI at the Cue test for WT and TgQBP1 mice. WT mice significantly reduce its average freezing when the acoustic cue is not present (Trial vs ITI, p=0.011), while TgQBP1 did not. Moreover, WT mice showed a trend to exhibit higher average freezing at trials than TgQBP1 mice (p=0.078).

**B)** Correlation and fit to a linear regression of freezing percentage with time in context extinction. The average per-minute freezing percentages recorded during the three contextual fear tests (24h, 96h, and 8d) for WT mice (blue) and TgQBP1 mice (orange), along with the results of the linear correlation analysis for both groups [WT: r=-0.5221 R^2^=0.2726 p=0.045 / TgQBP1: r=-0.0375 R^2^=0.0014 p=n.s.] are plotted.

**C)** Comparison of the total time of fear expressed by the animals in the extinction of the conditioning to the aversive context: at 24h, 4 days and 8 days after acquisition. Analyzing each group separately, only WT mice showed extinction over test days [F_(2,24)_=3,975 p=0,032] and there is a significant trend between 24-96h (p=0,081), while TgQBP1 mice did not extinguish [F_(2,21)_=0,9760 p=n.s.].

**D)** Results from Elevated plus maze to compare explorations in basal versus fear conditions. i) Considering fear conditions, these results show that there are no differences between groups and that they explore both arms with the same frequency. ii) Exploration in closed arms between conditions revealed that both groups change their explorations after fear conditioning (p<0.001), decreasing its frequency (WT p<0.001 and TgQBP1 p=0.001).

**E)** Results from Elevated plus maze to compare the percentage of entries in basal versus fear conditions. **i)** Considering fear conditions, these results show that both groups significantly vary their percentage of entries (p<0.001), increasing their percentage in closed arms (WT p<0.001 and TgQBP1 p=0.004). **ii)** Entries in closed arms between conditions showed that there is an interaction effect (Group x Condition p=0.041), with TgQBP1 mice reducing their entries after fear conditioning and WT mice increasing them.

For comparisons between independent groups: *p<0.05, **p<0.01, ***p<0.001, trends 0.05 ≥ #p<0.09; for comparisons between dependent groups: +p<0.05, ++p<0.01, +++p<0.001, trends 0.05 ≥ #'p < 0.09.

**Figure S5. Effect of age and gender in aversive memory and extended PTSD-like protocol results.**

**A)** Effect of gender in the adult WT mouse on aversive conditioning. In the acquisition phase, the two groups significantly learn conditioning with trials (T) and there were no differences between them. However, the percentage of total fear in the review of the aversive context at 24 h reflected that adult males remember significantly worse the fear with respect to adult females (p=0.045), which is maintained in the review of the context at 8 days (p=0.009).

**B)** Effect of sex in the younger WT mouse on aversive conditioning. In the acquisition phase, the two groups significantly learned conditioning with trials (T) and there are no differences between them. The absence of differences was maintained in the context review at 24 h, reflecting that there is no sex difference in the younger animals.

**C)** Effect of age on the consolidation of aversive memories in WT mouse males, where both groups significantly learned the conditioning with acquisition (p<0.001. However, in the review of context fear at 24 h and 96 h the younger WT mouse showed significantly more fear than the adult (p=0.061 and p=0.021 respectively).

**D)** Effect of age on the consolidation of aversive memories in females of the WT mouse, showing that during acquisition both groups significantly learned the conditioning (p<0.001) and there were no significant differences between age groups in the review of the context at 24 h and 72 h. However, adult female WT mice showed more freezing than younger female mice in the context review at 8 d post acquisition (0,004).

**E)** Percentage of fear in the cue test (conditioned stimulus recall), showing the Inter-trial (ITI) phase of the test. Both groups are able to discriminate the fear to the acoustic cue (fear increases just as cue is present), but it is more enhanced in the WT mice. There are no differences between groups at the post phase of the test.

**F)** Comparison of the average freezing percentage in the extinction days (aversive context recall) at 24h, 96h, 8 days and 14 days after acquisition. Analyzing each group separately, both groups significantly extinguished their fear to the conditioned context [WT: F_(4,43)_=6.257 p=0.009] / TgQBP1: F_(4,34)_=35.75 p<0.001], but only TgQBP1 mice showed significantly differences between days [Post-24h (p=0.018), 24-96h (p=0.004) and 96h-8d (p=0.058)].

**G)** Correlation and fit to a linear regression of freezing percentage with time in context extinction. The mean per minute of the freezing percentage is shown in the fear context recall test (Post acquisition, 24h, 96h, 8 days and 14 days) for the WT mouse (blue) and tgQBP1 mouse (orange). Also the result of the linear correlation of the data for both groups is shown [WT: r=-0.6342 R^2^=0.4023 p=0.007 / TgQBP1: r=-0.8426 R^2^=0.7100 p<0.001].

**H)** Total ingested volume (sucrose+water) in the sucrose preference test. The data revealed that WT mice ingested significantly more total volume than TgQBP1 mice (p=0,011). All data are displayed (colored circle).

For comparisons between independent groups: *p<0.05, **p<0.01, ***p<0.001, trends 0.05 ≥ # < 0.09; for comparisons between dependent groups: +p<0.05, ++p<0.01, +++p<0.001, trends 0.05 ≥ #' < 0.09.

**Figure S6: Additional co-immunoprecipitation results and amyloid characterization of hippocampal cytosolic fractions.**

**A)** Depiction of the strong extraction protocol for hippocampal homogenization. After the addition of detergent-containing buffer, the material is sonicated to maximize protein extraction. After one hour of incubation shaking on ice, the first initial low-speed centrifugation of the homogenate is performed, to yield a soluble fraction (cytosol content, S1) and an insoluble pellet (containing cell membranes and debris, P1). The S1 supernatant is subjected to cold ultracentrifugation to separate soluble proteins (supernatant, S2) from aggregated proteins (pellet, P2).

**B)** Native PAGE followed by Western blot detection with an anti-CPEB3 antibody in hippocampal (left panel) and remaining brain (right panel) extracts. Two distinct CPEB3-reactive bands are observed. Molecular weight markers are not indicated, as under native conditions proteins migrate according to their size, shape and intrinsic charge, and accurate molecular weight estimation is hindered by differences in conformation and folding.

**C)** Immunoblotting using an anti-GFP (left panel) and anti-CPEB3 (right panel) antibody of GFP-Trap Co-IP samples from kidney extracts. GFP bands are present on kidney extracts (positive control), but no CPEB3 signal is detected (serving as negative control due to its negligible expression in this tissue). Molecular weight markers are not indicated, as under native conditions proteins migrate according to their size, conformation, and intrinsic charge; consequently, accurate molecular weight estimation is hindered by the complex interplay of these factors rather than size alone.

**Figure S7: Differential proteomic analysis across tissues and experimental groups.**

Volcano plots illustrate the distribution of protein abundance changes (log2 fold change, x-axis) against statistical significance (-log10 adjusted p-value, y-axis). Panels A-D represent comparisons between treated and control groups in the hippocampus (A for females, B for males) and kidney (C for females, D for males). The horizontal dashed line indicates the significance threshold (FDR < 0.05). Red points (highlighted in panel C) identify significantly upregulated proteins in the kidney of treated females, while gray points represent proteins that did not meet the significance criteria.

**Figure S8: Additional biochemical studies showing differences among fractions and conditions.**

**A)** Aggregation kinetics of the fractions extracted from hippocampal S1-P1 after learning monitored by thioflavin T fluorescence intensity. The graphs corresponding to the first fractions of the extraction are reflected, where there are hardly any differences in aggregation in the cytosolic fraction (S1, upper panel), but there are differences in the insoluble fraction (P1, lower panel).

**B)** Immunodot blot of the insoluble fractions of the extracted hippocampi after learning, revealed with the conformational antibodies A11/OC and CPEB3 as control. The number of samples analyzed is reflected at the bottom: R refers to mouse and the number refers to the number of samples analyzed for it. Aβ and BSA were used as positive and negative control respectively.

**C)** Quantification of immunodot blot samples with Image J software for each developer used. The statistical analysis shows that there are no differences between groups in reactivity against A11/OC conformational antibodies, although there is a significant difference in the development with CPEB3 in the P1 fraction (p=0.007), where there is significantly more protein in the samples corresponding to the TgQBP1 mouse. Quantification of Aβ and BSA were used as positive and negative control respectively.

***Extended materials and methods***

**Analysis of hippocampal samples by Thioflavin-T fluorescence intensity (ThT)**

The assay was performed on Corning-384 plates, maintaining a final 20 μL volume: 1/6 dilution of the hippocampal extracts was analyzed. A continuous measurement was performed during 7 hours, programming the measurements every 120 seconds with a gain per well of 1300 at the FLUOStar OPTIMA Microplate Reader (BMG LABTECH), using two filters: a 440nm for excitation and 490 nm for emission. Buffer with the same amount of ThT was used as a control for fluorescence background.

**Immunodot blot assay**

This technique was used for the detection of the amyloid conformation and also the identification of the CPEB3 protein in their native state immobilized in a nitrocellulose membrane. We have analyzed their binding to antibodies that recognize specific structural conformations ^2^: A11 antibody, which detects the existence of prefibrillar (toxic) oligomers, and OC antibody, which detects fibrillar oligomers and mature fibers. Also we have used an anti-CPEB3 antibody specific for the sequence of this protein (Abcam, ab10883). Extracted hippocampal samples where diluted 1/6 and then 2 µl were placed on a 0.45 µm nitrocellulose membrane (Amersham Protran). The membrane was blocked using 10% fat-free milk (Blotting-Grade Blocker non-fat dry milk, Bio-Rad) and subsequently incubated with the corresponding primary antibody: A11 antibody diluted 1:2000 (Invitrogen, ref: AHB0052), OC antibody diluted 1:2500 (Millipore, AB2286) and CPEB3 antibody diluted 1:1000 (Abcam, ab10883). A fluorescently labeled anti-rabbit secondary antibody (IRDye 680RD, Li-Cor) was used for development on an Odyssey® CLx apparatus (LI-COR). Pre-fibrillar oligomers and fibrillar species of Aβ42 was used as positive control.

**Semi-denaturing detergent agarose gel electrophoresis (SDD-AGE)**

Samples of 50 µM hCEPB3 IDR (variable final volume, 50-100 μL) incubated at different times and in the presence/absence of different inhibitors were mixed with DNA loading buffer and 2% SDS was added. A total of 10-15 μL of the mixture was loaded onto 1.5% agarose gels (TAE 1x 0.1% SDS) and the electrophoresis was performed at 70 V for 2 h at 4 °C. Proteins were then transferred to a nitrocellulose membrane filter using a Trans-Blot device (Bio-Rad) and incubated with anti-OC (1:2500, Millipore) and anti-His tag (1:2000, Novagen) antibodies. Finally, the membrane was incubated with the LI-COR antibodies as described for the western blots and dot blots.

**Activity cage and Open Field (OF)**

The spontaneous motor activity of the animals was measured with an actimeter (VersaMax Legacy Open Field activity box, Omnitech Electronics), connected to a VersaMax analyzer and the VersaDat software. Two mice were placed *per* activity cage and the measurement was set up in five cycles of 1 minute each, in a two-day protocol. Animals were habituated to the room for 30 minutes before the experiment and the cage was cleaned with ethanol before and between animals. Same protocol was used for Open field test, using one mice *per* cage and it was set up in two cycles of 5 minutes each, done in a single day.

**Elevated plus maze (EPM)**

The testing apparatus consists of four black polypropylene arms (Coulbourn Instruments, Whitehall, PA), with two "open" arms and two "closed" arms (30 cm high walls); all 10 cm with in width, 50 cm long and placed at a height of 55 cm. At the beginning of each test, the animals were allowed to get used to the experimental room for 30 minutes and, at the beginning of the procedure they were placed in the center of the apparatus in front of an open arm. The animals were allowed to explore the apparatus for 5 min. The video signal was digitized and analyzed with the EthoVision XT 8.5 software (Noldus Information Technology, Leesburg, VA). An open arm entry was considered when the center of the mouse is in one of the open arms. The device was cleaned with ethanol before and between animals.

**Sucrose preference test (SPT)**

This test is based on a two-bottle decision where animals choose between drinking water or sugar water (reward), which measures their anhedonia after exposure to severe stress ^3^. We have implemented the protocol described in ^4^. This protocol consists of a first habituation of three days during which water is administered to the animals in two different bottles, followed by another two days where only sugar water (+ 2% sucrose) is administered. The animals are then deprived of water and food for 18 hours, after which a sucrose preference test is performed during the animals' active (dark) phase. It consisted in measuring liquid intake for two hours, where bottles were interchanged after the first hour to prevent side bias. Sucrose preference was calculated as the proportion of sucrose intake relative to the total fluid intake.

**Co‑immunoprecipitation (CoIP) – additional information**

Protein Electrophoresis- For SDS‑PAGE, protein extracts were mixed with LDS NuPAGE sample buffer (Thermo Fisher, Waltham, MA, USA; Cat. #NP0008) and heated at 95 °C for 2 minutes. Electrophoresis was performed on 4–20% Mini‑PROTEAN® TGX Stain‑Free™ Protein Gels (Bio‑Rad, Hercules, CA, USA; Cat. #4561093) at 200 V for 45 minutes in Tris‑Glycine‑SDS running buffer (Bio‑Rad, Hercules, CA, USA; Cat. #1610772).

## For native PAGE, proteins were extracted using native cell lysis buffer (Abcam, Cambridge, UK, ab156035) and preserved in Tris-Glycine sample buffer (Thermo Fisher, Waltham, MA, USA, LC267). Gel electrophoresis was performed using 4–20% Mini-PROTEAN® TGX Stain-Free™ Protein Gels (Biorad, Hercules, CA, USA) at 200V in Novex Tris-Glycine buffer (Thermo Fisher, Waltham, MA, USA, LC2672) for 2 hours in ice.

Western Blotting - Protein transfer was carried out using the Trans‑Blot® Turbo™ Transfer System (Bio‑Rad, Hercules, CA, USA; Cat. #1704270) at 25 V for 10 minutes. Proteins were transferred to 0.45 µm nitrocellulose membranes (Bio‑Rad, Hercules, CA, USA; Cat. #1620115), which were then blocked for 1 hour in 5% BSA in PBS containing 0.05% Tween‑20. Membranes were incubated overnight at 4 °C with primary antibodies (1:1000 dilution) [CPEB3 Polyclonal Antibody (Thermo Fisher, Waltham, MA, USA, Cat. MA5-15256 Ref: 12669-1-AP); GFP (D5.1) Rabbit Monoclonal Antibody (Cell Signaling, 2956S)], washed twice with PBS‑Tween, and incubated with the appropriate secondary antibodies (1:5000 dilution) for 1 hour at RT [Goat Anti-Rabbit IgG, H&L Chain Specific Peroxidase Conjugate (MERCK (Darmstadt, Germany, Cat. 401353)]. Signal detection was performed using Clarity Max™ Western ECL Substrate (Bio‑Rad, Hercules, CA, USA; Cat. #1705062) and visualized by chemiluminescence with the ChemiDoc™ Touch Imaging System (Bio‑Rad, Hercules, CA, USA).

**Mass spectrometry**

Protein pellets were resuspended with lysis buffer to 2.5% sodium dodecyl sulfate (SDS), and 50 mM triethylammonium bicarbonate (TEAB).Samples were reduced and alkylated by adding 10 mM tris(2-carboxyethyl)phosphine (TCEP) and 10 mM chloroacetamide (CAA) for 1h at 37ºC. Protein digestion in STRAP columns (PROTIFI, Farmingdale, NY) was performed following the manufacturer's instructions with minor changes. Each sample was digested at 37°C overnight using trypsin bovine (Sequencing Grade Modified Trypsin, Promega) in a trypsin:protein ratio of 1:15 and was cleaned with a StageTip C18 (SPE EmporeTM 47mm extraction disks, SUPELCO) prior to LC-ESI-MS/MS analysis.

After desalting, tryptic peptides were quantified by QUBIT Fluorometric Quantitation (Thermo Scientific, Rockford, IL, USA).Peptides (500ng per sample or ½ of total sample) were subjected to 1D-nano LC ESI-MS/MS (Liquid Chromatography Electrospray Ionization Tandem Mass Spectrometric) analysis using an Ultimate 3000 nano HPLC system (Thermo Fisher Scientific) coupled online to an Orbitrap Exploris 240 mass spectrometer (Thermo Fisher Scientific). Peptides (5 μL corresponding to 500ng of peptides or ½ of total sample) were injected on a C18 PepMap trap column (5 µm, 300 µm I.D. x 2 cm, Thermo Scientific) at 20 µL/min, in 0.1% formic acid in water, and the trap column was switched on-line to a QFC Column (75μm x 250 mm C-18A, Montara BIOLABS) at 50°C, at a flow rate of 250 nL/min using a 90 min gradient ranging from 2 % to 95 % mobile phase B (mobile phase A: 0.1% formic acid (FA); mobile phase B: 80 % acetonitrile (ACN) in 0.1% FA). The loading solvent was 2 % ACN in 0.1 % FA and injection volume was 5 µl.

Data acquisition was performed using a data-dependent (DDA) top-20 method, in full scan positive mode, scanning 375 to 1200 m/z. Survey MS1scans were acquired at a resolution of 60,000 at m/z 200, with Normalized Automatic Gain Control (AGC) target adjusted to 300 (%) and a maximum injection time (IT) of 45 ms. The top 20 most intense ions from each MS1 scan were selected and fragmented via Higher-energy collisional dissociation (HCD). Resolution for MS2 spectra was set to 15,000 at m/z 200, with AGC target of 100 % and a maximum ion injection time of 80 ms. Isolation of precursors was performed with a 1 m/z window, dynamic exclusion was set to 45s and the HCD collision energy was set to 32. Precursor ions with single, unassigned, or six and higher charge states from fragmentation selection were excluded.

Raw files were processed using Proteome Discoverer (PD) version 3.1 (Thermo Fisher Scientific). MS2 spectra were searched against engine Mascot (v3.1), and a target/decoy database containing “Mus musculus” protein sequences downloaded from Uniprot Knowledgebase Database. Static modification carbamidomethylation on cysteine (+57.021464 Da) were considered, while oxidation of methionine residues (+15.994915 Da), Acetyl (Protein N-term) (+42.010565 Da) and Gln->pyro-Glu (N-term Q) (-17.026549 Da) were set as a dynamic modifications. A maximun number of 2 tryptic misscleavages was allowed. Peptide precursor and MS/MS mass tolerance was set to10 ppm and 0.02 Da, respectively. The false discovery rate (FDR) for proteins, peptides, and peptide spectral matches (PSMs) peptides was set to 1%.

The mass spectrometry proteomics data have been deposited to the ProteomeXchange Consortium via the PRIDE partner repository with the dataset identifier PXD078931 ([http://www.ebi.ac.uk/pride/archive/projects/PXD078931](http://www.ebi.ac.uk/pride/archive/projects/PXD000000)).

**Proteomic differential analysis**

Protein identification data were retrieved from individual Proteome Discoverer exports in XLSX format, utilizing the “Proteins” sheet for each sample. Initial filtering at the input level retained only high-confidence proteins with an FDR (q-value) < 0.01, excluding common contaminants and reverse/decoy sequences. Protein abundances were log2 transformed and normalized via median-centering to ensure inter-sample comparability.

Differential expression analysis was performed by comparing the control group against each treatment group: Hpp Female, Hpp Male, R Female, and R Male. To ensure data robustness, only proteins identified in at least two biological replicates per group were considered for downstream analysis. Statistical inference was conducted using the R package limma (v3.66.0), applying empirical Bayes moderation with the trend = TRUE and robust = TRUE parameters.

To account for the dependency between protein variance and the number of observations, the R package DeqMS (v1.28.0) was employed. A spectrum-number-dependent adjustment was applied via spectraCounteBayes, using a consolidated count per protein (calculated as the median of PSMs, peptides, and unique peptides per Accession, rounded to the nearest integer with a minimum value of 1). Multiple testing correction was performed using the Benjamini-Hochberg method on the sca.P.Value generated by DeqMS. Proteins with a False Discovery Rate (FDR) < 0.05 were considered significantly differentially expressed.

**Congo-red binding**

For the spectroscopic test with CR, a working solution at 40 μM in buffer (5 mM potassium phosphate, 150 mM NaCl, pH 7.4) was prepared and filtered immediately before use. 5 μL of protein (10 μM, incubated until the corresponding time in each case) was added to 5 μL of the stock CR solution, so that a final ratio of 1:2 (protein:CR) is maintained. The UV-visible spectrum was recorded in a spectrophotometer (BioSpectrometer, Eppendorf) between 400 and 700 nm, collecting the absorbance values at 480 and 540 nm to subsequently calculate the concentration of bound CR ^5,6^.

***References for supplementary material***

1.	Crawley, J. N. Behavioral phenotyping of transgenic and knockout mice: Experimental design and evaluation of general health, sensory functions, motor abilities, and specific behavioral tests. *Brain Res.* **835**, 18–26 (1999).

2.	Kayed, R. *et al.* Fibril specific, conformation dependent antibodies recognize a generic epitope common to amyloid fibrils and fibrillar oligomers that is absent in prefibrillar oligomers. *Mol. Neurodegener.* **2**, 1–11 (2007).

3.	Liu, M. Y. *et al.* Sucrose preference test for measurement of stress-induced anhedonia in mice. *Nat. Protoc.* **13**, 1686–1698 (2018).

4.	Fernández de Sevilla, M. E. *et al.* Insulin-like growth factor I mitigates post-traumatic stress by inhibiting AMP-kinase in orexin neurons. *Mol. Psychiatry* **27**, 2182–2196 (2022).

5.	Wurth, C., Guimard, N. K. & Hecht, M. H. Mutations that reduce aggregation of the alzheimer℉s Aβ42 peptide: An unbiased search for the sequence determinants of Aβ amyloidogenesis. *J. Mol. Biol.* **319**, 1279–1290 (2002).

6.	Hervás, R. *et al.* Common features at the start of the neurodegeneration cascade. *PLoS Biol.* **10**, e1001335 (2012).
